# A review of the North American genus *Epimartyria* (Lepidoptera, Micropterigidae) with a discussion of the larval plastron

**DOI:** 10.3897/zookeys.183.2556

**Published:** 2012-04-19

**Authors:** Donald R. Davis, Jean-François Landry

**Affiliations:** 1 Department of Entomology, National Museum of Natural History, Smithsonian Institution, P.O. Box 37012, MRC 105, Washington, D.C. 20013-7012, USA; 2 Agriculture and Agri-Food Canada, Eastern Cereal and Oilseed Research Centre, C.E.F., Ottawa, Ontario K1A 0C6, Canada

**Keywords:** Distribution, DNA barcodes, genital morphology, larval morphology, plastron

## Abstract

The indigenous North American micropterigid genus *Epimartyria* Walsingham,1898 is revised. Three species are recognized, including *Epimartyria auricrinella* Walsingham, 1898 which occurs widely over much of the northeastern United States and Canada, a new species, *Epimartyria bimaculella* Davis & Landry from northwestern United States and Canada, and *Epimartyria pardella* (Walsingham, 1880) from northern California to northern Oregon. The larva of *Epimartyria auricrinella* is described in detail, supplemented with illustrations of the external structure of the larval integument. The larval plastron is described and illustrated for *Epimartyria*, and this is compared with the plastrons of *Neomicropteryx* Issiki, 1931 and *Micropterix* Hübner, 1825. COI barcode sequences show that the three species are genetically distinct, congruent with morphological differences. Marked haplotype divergence within some *Epimartyria auricrinella* populations appears to be unrelated to morphology, geography or phenology.

## Introduction

The archaic moth family Micropterigidae constitutes the only member of the suborder Zeugloptera and is one of three extant families whose adults are partially characterized as possessing articulated mandibles and in having never developed a coilable proboscis (Kristensen 1998). The oldest fossil remains of Micropterigidae are known from lower Cretaceous Lebanese amber of ~ 140 mya (Whalley 1977, 1978). Approximately 21 genera and 160 species of Micropterigidae are now known, with more than 100 additional species recognized but not described (Nieukerken et al. 2011). The family is widely distributed , with no records yet reported from Amazonia, or equatorial Africa. However, the recent discovery of two undescribed genera from lowland (and montane) Costa Rica indicates that the family can also occur in more equatorial, tropical rainforests.

Micropterigidae typically occur in humid habitats where their larvae frequently feed on foliose liverworts or possibly on fungi within rotten logs or soil (Gibbs (2010). Heath (1976) reported larvae of *Micropterix* Hübner, 1825 at depths down to 10 cm. in loose soil. Occasionally fresh as well as decaying angiosperm leaves may be consumed by larval *Micropterix*. Lorenz (1961) reared larvae of *Micropterix calthella* (Linnaeus, 1761) on decayed plant detritus as well as upon fresh leaves of *Veronica agrestis* L. Carter and Dugdale (1982) found that successful rearing of two species of British *Micropterix* was dependent upon a supply of fresh, photosynthetic angiosperm tissue, particularly chickweed (*Stellaria media* (L.). The number of larval instars is known to vary between 3 in *Epimartyria* (Tuskes and Smith, 1894) and 4 in *Micropterix* (Klausnitzer et al, 2002), *Kurokopteryx* Hashimoto, 2006 and *Neomicropteryx* Issiki, 1931, (Hashimoto 2006). Adult Micropterigidae are known to feed on plant pollen from a broad range of angiosperm families (Zeller-Lukashort et al. 2007). Members of the southwestern Pacific *Sabatinca* group have also been reported feeding on fern spores (Kristensen 1998, Gibbs 2010). Adults of a few new species of Micropterigidae have been recently discovered in Costa Rica feeding on fern spores (Wagner and Davis in prep.).

Major portions of the larval integument of *Epimartyria auricrinella* have been found to be densely covered with minute, irregularly shaped micropapillae (Davis 1987).Because theminute size and distribution of these cuticular structures closely resemble those of other insects known to inhabit aquatic or occasionally flooded habitats, it is believed that portions of the integument of *Epimartyria* may also serve in assisting respiration as has been demonstrated in those species (Thorpe 1950, Hinton 1969, 1976). These specializations are discussed further under the larval morphology of *Epimartyria auricrinella*.

Five monophyletic lineages have been determined within the Micropterigidae based on analysis using the 16S rRNA gene (Kobayashi et al. 2000, Gibbs et al. 2004). *Epimartyria* is a member of the northern hemisphere group which is represented by five genera in Japan: *Issikiomartyria* Hashimoto, 2006, *Kurokopteryx*, *Neomicropteryx*, *Palaeomicroides* Issiki, 1931, and *Paramartyria* Issiki, 1931, with a single genus each known from Vietnam (*Vietomartyria* Hashimoto & Mey, 2000), and North America (*Epimartyria*) (Gibbs 2010).

## Material

Specimens examined in this study are deposited in the following institutions:

**BMNH **The Natural History Museum (formerly the British Museum (Natural History), London, United Kingdom.

**BIO **Biodiversity Institute of Ontario, University of Guelph, Ontario, Canada.

**CZC** Collection of Christof Zeller-Lukashort, Thalgau, Austria.

**CNC **Canadian National Collection of Insects, Arachnids and Nematodes, Agriculture and Agri-Food Canada, Ottawa, Ontario, Canada.

**ONPS **Olympic National Park Service Collection, Port Angeles, Washington, USA.

**UCB** Essig Museum of Entomology, University of California, Berkeley, California, USA.

**USNM **Collections of the former United States National Museum, now deposited in the National Museum of Natural History, Smithsonian Institution, Washington, D.C., USA.

**WSDAC** Washington State Department of Agriculture Collection, Olympia, Washington, USA.

## Methods

### Specimen preparation

Genitalic dissections were cleared by heating in hot 10% KOH for ~ 30 minutes, and subsequently cleaned and stained with either 2% chlorazol black E or mercurochrome solutions. All genitalic illustrations were drawn from dissections temporarily stored in glycerine, which were later permanently embedded in Canada balsam or Euparal. Genitalic terminology follows Klots (1970) and Kristensen (1984b). Samples of alcohol-preserved larvae and pupae were gently washed in 409 ® detergent, then dried in a critical point drier, sputter coated with 20–25 gold palladium 60:40 alloy, and photographed with an Amray 1810 scanning electron microscope.

### Molecular analysis

DNA barcodes were produced at the Canadian Centre for DNA barcoding at the Biodiversity Institute of Ontario, University of Guelph following standard protocols (Hebert et al. 2003; Floyd et al. 2009). 1–2 legs were removed from adult moths for DNA extraction. All Voucher data, images, sequences, and trace files are publicly available on the Barcode of Life Database (BOLD) (Ratnasingham and Hebert 2007). Sequences were also deposited in GenBank. Sample IDs, Barcode IDs, and GenBank Accession numbers are listed in Appendix 1. Neighbour-Joining (NJ) trees for all barcode data were constructed using the quicktree algorithm (Howe et al. 2002) and under the Kimura two-parameter (K2P) model of base substitution (Kimura 1980). Genetic distances were estimated with MEGA 5.05 (Tamura et al. 2011) using the K2P model. Maximum parsimony (MP) analyses was performed with PAUP* 4.0d100 (Swofford 2003) on selected sequences representing distinct haplotypes. Only full-length barcode sequences without ambiguous sites were analyzed. Heuristic searches for MP analysis were carried out with all positions equally weighted and under the tree bisection-reconnection (TBR) swapping algorithm with 100 random addition sequences. Bootstrapping of 1000 replicates was conducted under the parsimony criterion with the default setting starting with a random seed and the TBR branch-swapping algorithm. Bremer support values were calculated using Treerot v.3 (Sorenson and Franzosa 2007). Haplotype diagrams were constructed in TCS 1.21, with a 95% confidence limit for parsimony (Templeton et al. 1995). Shorter sequences or those with ambiguous bases were excluded from the haplotype analysis.

## Systematic account

### Epimartyria

Walsingham

http://species-id.net/wiki/Epimartyria

Epimartyria Walsingham, 1898: 161.– Kearfott in Smith 1903: 125.– Dyar 1903: 581.– Meyrick 1912: 3.– Forbes 1923: 63.- McDunnough 1939: 2.– Davis 1983: 5; 1987: 341.– Kristensen 1984b: 97.– Nye and Fletcher 1991: 113.– Poole 1996: 716.– Hashimoto 2006: 98.
Micropteryx Hübner.– Forbes 1923: 64 (subgenus *Epimartyria* Walsingham).

#### Type species.

*Micropteryx pardella* Walsingham, by original designation.

#### Diagnosis.

*Epimartyria* appears closely allied to the Asian genus *Paramartyria* as suggested by the similar elongate process arising from the inner base of the male valvae (Fig. 78) and by similar larval chaetotaxy (Hashimoto 2006). More significantly, close affinities of these two northern genera were also indicated from the molecular study initiated by Kobayashi et al. (2000) and Gibbs et al. (2004), based on the 16S rRNA gene. At least one species of the Asian genus *Vietomartyria*, *Vietomartyria nankushana* Hirowatari & Hashimoto (Hirowatari et al. 2009), also posseses a similar basal process on the valva, as pointed out by one reviewer. The forelegs of *Vietomartyria* also lack an epiphysis as do two species of *Epimartyria*. *Epimartyria* differs from all other micropterigid genera in possessing a deeply divided phallus, and from *Paramartyria* in particular by possessing a pair of lateral projections near the apical one third of the distal phallus and in having tergum X divided into dorsal and ventral processes (Hashimoto 2006). Hashimoto (2006) also mentioned the presence of an epiphysis in *Paramartyria* as one feature that distinguishes the latter from *Epimartyria*. Two of the three species of *Epimartyria* lack an epiphysis, but an epiphysis is present in *Epimartyria pardella* (Fig. 17).

#### Adult.

*Head* (Figs 13–15): Vestiture entirely hairy, scales erect and piliform with acute apices. Antenna (Figs 18–20) 0.75–0.9× length of forewing, slightly longer in male; pedicel enlarged, ~ 1.5× length of first flagellomere; flagellum moniliform, with 46–58 flagellomeres in male, 38–47 in female; flagellomeres mostly sparsely covered with long, piliform scales which exceed the length of their supporting flagellomere; basal 2–3 flagellomeres in male and 5–7 in female covered dorsally with moderately broad scales; a pair of large ascoid sensilla, opposite one another, with ~ 11–16 elongate, curved, sensory branches (Fig. 18) on each flagellomere; a single irregularly shaped and often bilobed multiporus sensillum placodeum (Faucheux 1997) arising between the ascoid sensillae from a shallow pit near the ventral anterior margin of the flagellomere. Compound eyes reduced, interocular index (Davis 1975) ~ 0.35–0.37; interfacetal setae absent. Ocelli present, base moderately elevated. Labrum approximately pentagonal, length ~ 2× that of clypeus. Mandible elongate triangular in form; distal edge truncate. Maxillary palpus elongate, 5-segmented, with main flexions between segments 1 and 2 and between 3 and 4; length ratio from basal segment 1: 1:2.7:2.7:0.9. Labial palpus short, total length ~ equal to that of basal segment of maxillary palpus; 2- segmented; sensory pit (organ vom Rath) present distally on apical segment enclosing numerous sensillae; apices of most sensillae terminating in a cluster of ~ 2- 5 minute acute lobes (Figs 21–22). Proximal prelabial sclerite slender, crescentiform; distal prelabial sclerite broadly triangular. Occipital sulcus incomplete but distinct laterally.

*Thorax*: Scales of mesonotum broad, appressed. Metanotum mostly naked except for a few long, piliform scales. Tegulae rather sparsely covered with long piliform scales. Forewing length: 4.2–5.5 mm; forewing (Fig. 16) with humeral vein present; Sc deeply bifurcate; R simple; Sc-R crossvein present near fork of Sc; Rs with 4 veins; Rs3–4 fused to ~ basal 1/3; accessory cell present; M with 3 branches; 1A and 2A fused over distal half; 3A extending across base of moderately small jugal lobe. Wing scale morphology of the primitive, generally non-glossatan type (Kristensen and Simonsen 2003) consisting of fused dorsal and ventral surfaces (without internal chambers), and with a herringbone pattern formed by oblique-longitudinal crests overlying a dense layer of transverse microribs (Figs 28–31). Hindwing venation similar to forewing except with Sc and R fused; 1A and 2A completely fused; anal crossvein connecting to CuP near distal 2/3; scales over distal third of hindwing dark fuscous and nearly as broad and iridescent as in forewing; scales gradually becoming more slender, gray, and without iridescence over basal 2/3. Legs (Fig. 17) with tibial spur pattern of 0–0-4; a short epiphysis ~ 1/3 the length of tibia arising slightly beyond its midlength present in *Epimartyria pardella*; epiphysis absent in *Epimartyria auricrinella* and *bimaculella*; pretarsus (Figs 24–27) consisting of a pair of strongly curved claws; a lateral pair of pad-like pulvilli densely covered with long spinose setae; a median arolium with apical surface densely lined with minute grooves (Fig. 27); pseudempodial seta (Fig. 25) with longitudinal grooves.

*Abdomen*: Cuticle dark brown, sparsely covered with long, piliform scales. A pair of glands present, opening on sternum V in both sexes (Philpott 1925); glands similar to those present in *Paleomicroides*, *Paramartyria*, and *Neomicropteryx* in not protruding and possessing a narrow slit-like opening within a smooth, hyaline area (Kristensen 1984a).

*Male genitalia*: Tergum X (uncus) ~ half the median ventral length of IX; apex deeply divided nearly half its length into two broad lobes. Sternum X (venter X) variously bilobed, with or without short lateral lobes. Segment IX a completely sclerotized ring, with dorsal median length ~ 1/6 of ventral length. Sternum IX (vinculum) a broad plate with subparallel lateral margins; anterior end as broad or broader than caudal end. Valva with a subacute to rounded apex; base of valva with a long digitate process from mesal surface. Medial plate (juxta) with a slender stalk-like base gradually expanding anteriorly to a small, flat, oval plate. Distal phallus divided into two slender branches ~ half the total length of phallus; shorter dorsal branch of phallus terminating in gonopore (phallotreme) with thickened radial folds; a pair of minute, acute spines present laterally near distal third of dorsal branch; apex of longer ventral branch densely covered with numerous minute flattened scutate processes with rounded apices directed basad; phallobase moderately inflated, as long as or slightly longer than divided branches.

*Female genitalia*: Abdominal segment IX a complete ring with mid dorsal length ~ 0.5–0.6× the mid ventral length. Segment X consisting of a pair of lateral, setose plates; cloaca ending terminally; X often telescoped into IX and VIII in repose. Apophyses absent. Genital chamber with thickened walls surrounding a variably shaped slerite; caudal end of sclerite furcate. Ductus spermatheca with a moderately enlarged, spindle-shaped reservoir (utriculus) located at varying distances along ductus. Corpus bursae gradually enlarging anteriorly, membranous, with four tridentaform signa equally spaced around middle of corpus bursae; enlarged bases of signa projecting externally beyond wall of corpus bursae, with spinose branches projecting internally.

#### Remarks.

For many years John Heath, formerly employed at the Experimental Research Station at Monks Wood in England, pursued research on the family Micropterigidae, resulting in about 20 papers on this group (Emmet 1987). Heath had partially completed a revision of the genus *Epimartyria*, but this was never published. We had not viewed a copy of this manuscript until our publication was in review. In his manuscript, Heath recognized an additional new species from New Jersey, based on specimens collected at Essex County Park by W. D. Kearfott. Our studies found no morphological justification for this species.

Because this is the first taxonomic revision of *Epimartyria*, there remain some gaps in our knowledge about their biology which cannot be answered with available material and evidence.

#### Key to species of *Epimartyria*

**Table:** 

1	Forewing without spots, uniformly dark fuscous with coppery to purplish luster (Fig. 1)	*Epimartyria auricrinella*
–	Forewingwith pale yellowish spots	2
2	Forewingwith 2 pale yellowish spots (Fig. 2); foretibia with epiphysis absent; caudal apex of male sternum X (gnathos) deeply divided, with apex of lobes acute (Fig. 82)	*Epimartyria bimaculella*
–	Forewingwith 4 pale yellowish spots (Fig. 3); foretibia with epiphysis present; caudal apex of male sternum X not deeply divided, with short, triangular, caudal lobes (Fig. 89)	*Epimartyria pardella*

### Epimartyria auricrinella

Walsingham

http://species-id.net/wiki/Epimartyria_auricrinella

**Figures 1–3.**
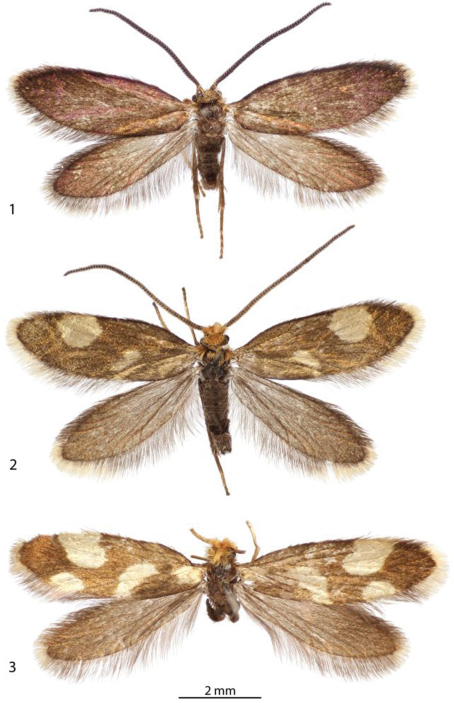
Adults**. 1** ♂, *Epimartyria auricrinella*,  (4.9 mm) Canada: Quebec **2** ♂, *Epimartyria bimaculella*  (5.5 mm) Holotype, Canada: British Columbia **3** ♀, *Epimartyria pardella* (5.5 mm) USA: California. (Forewing length in parentheses).

Figs 1 4–5 10 18 [Fig F9] [Fig F10] -32 33 [Fig F13] [Fig F14] [Fig F15] -59 74–80

Epimartyria auricrinella Walsingham, 1898: 162.– Kearfott in Smith 1903: 125.– Dyar 1903: 581.– Meyrick 1912: 6.– McDunnough 1939: 110.– Davis 1983: 5; 1987: 341.– Poole 1996: 716.– Djernaes 2011: 3.– Hashimoto 2006: 43.
Micropteryx Epimartyria auricrinella Walsingham.– Forbes 1923: 64.

#### Diagnosis.

Adult *Epimartyria auricrinella* are easily distinguished from those of the other members of *Epimartyria* in possessing uniformly dark fuscous forewings without the yellowish spots present in those species.

#### Adult

**Figures 4–9.**
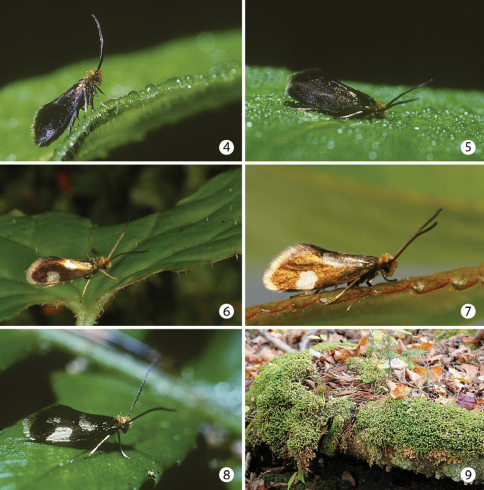
Adults and habitat. **4–5** *Epimartyria auricrinella*, at Lac Brûlé, Québec, 30 Jun 1997, ca 0700 hrs. on dewy *Solidago* leaf **6–7** *Epimartyria bimaculella* **6** at Washington, Olympic National Park, Hoh Rainforest Road, 22 Jun 2010 (photo by Zeller-Lukashort) **7** at British Columbia, Vancouver area, Belcarra, 24 May 2009, ca 1000 hrs (photo by Holden) **8** *Epimartyria pardella*, California, Redwood National Park, Gold Bluffs State Beach, Fern Canyon **9** Habitat, clump of the liverwort *Bazzania trilobata* at Lac Brûlé, Québec in which larvae of *Epimartyria auricrinella* were found.

(Figs 1, 4–5). *Head*: Vestiture light orange brown. Antenna with vestiture of scape and pedicel concolorous with head; scales of flagellum dark brown to fuscous. Labial palpus cream.

*Thorax*: Dark fuscous with coppery to purplish luster. Tegula concolorous with head. Forewing dark fuscous with coppery or golden to purplish luster dorsally, less iridescent ventrally; fringe paler, more gray. Forewing length: 4.2–5.6 mm. Hindwing with scales over distal third nearly as broad, dark fuscous and iridescent as in forewing; scales gradually becoming more slender, more gray, and less iridescent over basal 2/3; fringe gray. Legs medium to dark brown dorsally, light brown ventrally and at apices of tarsomeres; epiphysis absent.

*Abdomen*: Piliform scales uniformly brown dorsally and ventrally. Paired glands of sternum 5 with muscle for opening glands originating on anterior edge of sternum 6 and inserted into each gland duct just inside aperature; gland reservoir slightly larger and more ovoid in female, but surrounding layer of secretory cells better developed and 2–3× thicker in female (Djernaes 2011, Djernaes and Sperling 2011).

*Male genitalia* (Figs 74–78): Caudal lobes of tergum X broadly rounded. Caudal apex of sternum X deeply divided, with apex of lobes acute, recurved; a pair of short, lateral lobes present near base. Valvae moderately long, ventral length nearly half the maximum length of segment IX; apex subacute and bearing a short, slender, recurved spine; a short, triangular, rounded process arising midway from mesal surface; elongate basal process ~ 4/5 the length of valva; distal margin of valva variable within populations from slightly concave to convex (Figs 78a-d). Dorsal branch of phallus cylindrical and smooth.

*Female genitalia* (Figs 79–80): As described for genus. Caudal end of genital sclerite moderately furcate as in *Epimartyria bimaculella*; length of furcations ~ 0.2 that of relatively shorter, undivided base.

#### Larva

(33–59). Mature larva up to 5 mm in length. Body approximately hexagonal in cross section; color generally brown, lighter brown ventrally. Integument over dorsal half of body with a honeycomb-like surface of raised ridges (Figs 52, 58); integument of ventral half densely covered with micropapillae (Fig. 56) with an extensive plastron surface laterally (Fig. 52). Primary setae longitudinally ribbed, moderately slender, long, clavate.

*Head*: Prognathous and capable of being retracted into prothorax. Antenna elongate, slender, 3- segmented, arising posterior of clypeal margin and dorsal to stemmata; second segment the longest, ~ 2× the length of basal segment; all antennal segments without sensory setae except for elongate terminal spinose seta. Five stemmata present, arranged in a tight circle. Adfrontal sutures vestigial, not extending to vertex; adfrontal ridges similarly undeveloped. Ecdysial lines externally indistinct. Tubular spinneret absent; external opening of labial salivary gland circular, relatively large, diameter ~ equal to length of second segment of labial palpus. Cranial setae reduced in length and number and concentrated over anterior third of head; stemmatal setae absent; a single medial (M) seta arising midway between antennae, without homology in other Lepidoptera but possibly homologous to campaniform sensillum in Trichoptera larva (Kristensen 1998). Labrum with 6 pairs of primary setae and numerous spines along anterior margin; seta La 1 arising distad of anterior margin of labrum (Fig. 38). Mandible generally triangular in form with 3 acute cusps, the basal-most cusp the most reduced. Maxillary palpi relatively well developed, 3–segmented, with apical sensillae as in Fig. 46. Labial palpi reduced, 3 - segmented with minute apical segment bearing a long sensillum (Fig. 47). Intersegmental membrane between head and thorax covered with flattened, multidentate, scutate outgrowths (Figs 41–42).

*Thorax*: Prothorax with 7 primary tactile setae and 4 peg-like microsetae, the latter located along anterior margin of prothorax near the head- prothoracic fold; XD1 and XD2 greatly reduced to peg-like microsetae along dorso-anterior margin of prothorax below D2; L1 posterior to XD1; L2 below L1 and anterior to spiracle. MV1 and MV2 short, peg-like, below SV2 and closer to anterior margin of prothorax; MV2 about 2 × length of MV1. Subdorsal setae absent on all body segments. Meso- and metathorax with 5 primary setae and one microseta (SV2); L1 and 2 well developed and equal in size. Legs with 3 well defined segments and large pretarsal segment; 4- segmented including reduced coxa; pretarsal claw curved, elongate, ~ 1/3 the length of remainder of leg; axial spine at base of claw well developed; femur and trochanter fused, as well as tibia and tarsus; coxa with a bilobed and possibly eversible tactile vesicle located posterior-mesally near base of femur (Figs 48–49);

*Abdomen*: Segments 1–8 with 4 primary setae and 2 peg-like (L2) to spherical microseta (SV2); segment 9 with only D1 and L1; segment 10 with 2 microsetae, possibly representing D1 and L1. Spiracles peripneustic, located anteriorly in intersegmental fold on segments 1–8; spiracle raised to form a small dome with walls subdivided into ~ 10–12 fimbriated bands. Abdominal segments 1- 8 with short, fleshy, nonmuscular prolegs with rounded apices (Fig. 50); crochets absent in all genera of Micropterigidae.

#### Larval hosts.

Hepaticophyta: Lepidoziaceae: *Bazzania trilobata* (L.) S. Gray.

#### Pupa.

Unknown.

#### Biology

**Figures 10–11.**
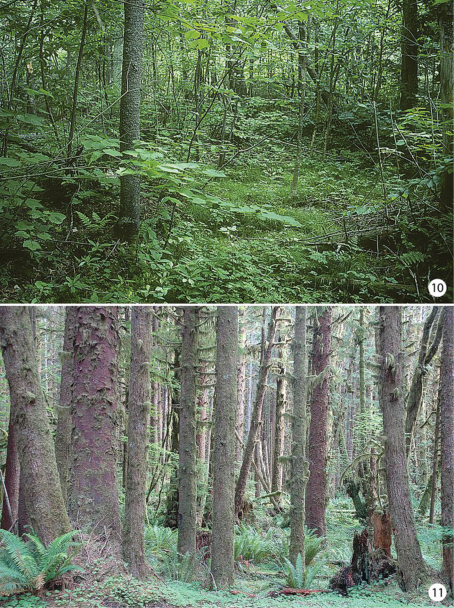
Habitats of *Epimartyria* **10** Swampy forest at Lac Brûlé, Quebec where larvae and numerous adults of *Epimartyria auricrinella* were collected **11** Douglas fir forest where adults of *Epimartyria bimaculella* were observed swarming around the ferns (photo by Zeller-Lukashort).

(Figs 9–10). The species occurs in shaded locations, in wet swampy woods, boggy ditches, or creek sides where leafy (moss-like) liverworts, the probable larval host, grow. Such habitats can be periodically or seasonally flooded. Larvae possess a plastron which indicates the capacity to live for short periods in a subaquatic environment or, at least in a habitat that is water-saturated. Adults are diurnal and are best obtained by gently sweeping the understory or clumps of liverworts (Landry and Landry 1992). They can be seen perched on low foliage during the day and can be active even in early morning after sunrise (Figs 1–2). Mating was observed in the afternoon between 1200–1700H (JFL pers. obs.). Larvae obtained (by JFL) by placing in a Berlese funnel clumps of the liverwort *Bazzania trilobata* collected on 3 September 2000 at Lac Brûlé (Quebec) yielded larvae of two different size classes (3.4 mm vs 1.8 mm overall body length). This supports the previous observations by Davis (1987) that larval development probably spans over two years, at least in the northern part of the range, although adults emerge every year. One larva was found on the tip of a liverwort leaflet at the same locality in early October when the air temperature was around 5°C. Adults generally begin to emerge in mid May in the southern part of their range (Georgia, North Carolina) with April 30 being the earliest date recorded (from southern Maryland near Washington, DC). Further north the flight period is gradually delayed, with adults in northern New York and all of Canada active during the summer between mid-June and mid-July.

#### Holotype.

♂**,** USA**:** North Carolina, 1884, H. K. Morrison, Type No. 35325, slide BM 8947 (BMNH).

#### Material examined.

CANADA: NOVA SCOTIA: Baddeck: 1 ♂, 23 Jun 1936; 1 ♂, 30 Jun 1936, T.N. Freeman, specimens # CNCLEP00077282–00077283, slide MIC1825 (CNC); Parrsboro: 1 (abdomen missing), 12 Jul 1944, J. McDunnough, (CNC). ONTARIO: Ottawa: 4 ♂, 19 Jun 1906, C.H. Young, slide USNM 16615 (USNM, CNC); 5 ♂, 20 Jun 1906, C.H. Young, slide USNM 34372, specimens # CNCLEP00077266–00077268, CNC slide MIC1822 (CNC, USNM); 1 ♂, 27 Jun 1906, slide USNM 98008 (USNM); 1 ♂, 12 Jun 1946, G.S. Walley, specimen # CNCLEP00077269 (CNC). Black Lake, N of Burgess Township: 1 ♂, 22 Jun 1974; 2 ♂, 1 ♀, 14 Jun 1975, J.A. Downes, specimens # CNCLEP00077277–00077280 (CNC). Moosonee: 1 ♀, 18 Jul 1934, G.S. Walley, specimen # CNCLEP00077288 (CNC). Orillia: 3 ♂, 26 Jun 1926; 1 ♂, 3 ♀, 2 Jul 1926, C.H. Curran, specimens # CNCLEP00077276–00077270–00077276, CNC slide MIC1823 (CNC). Thunder Bay: 1 ♀, Jul 1945, H. S. Parish. QUEBEC: Havre-Saint-Pierre: 4 ex., 3–17 Jul 2010, malaise trap, C. Bélanger. Gaspé Peninsula: Mont Albert: 2 ♂, 1 ♀, 18 July 1940, A. E. Brower, slides USNM 16621, 17501, SEM slide 18394 (USNM); 11 ♂, 1 ♀, 19 Jul 1940, 2♂, 22 Jul 1940; side Mt. Albert: 2 ♂, 2 ♀, wing slide USNM 16157, slides USNM 18396, 18408, 98007, SEM slide 18430, (USNM, CNC). Mansonville: 1 ♂, 18 Jun 1928, W.J. Brown, specimen # CNCLEP00077286, CNC slide MIC1824 (CNC). Ste-Agathe-des-Monts, Lac Brûlé, 46.0903°N, 74.26°W, 370 m: 11 ♂, 6 ♀, 26 Jun 1991, afternoon sweeping in swampy ditch with liverworts and mosses at edge of spruce forest, J.-F. Landry, specimens # CNCLEP00076615–00076631 (CNC); Lac Brûlé, 46.0909°N, 74.2756°W, 370 m: 9 ♂, 3 ♀, 8 Jul 1992, 2 ♂, 16 Jul 1992, day sweeping on shaded liverworts near boggy marsh, J.-F. Landry; specimens # CNCLEP00068799–00068800 (CNC, USNM); Lac Brûlé, 46.0903°N, 74.26°W, 370 m: 3 ♂, 2 ♀, 1 Jul 1993, afternoon sweeping liverworts and mosses, J.-F. Landry, specimens # CNCLEP00067565–00067569 (CNC); Lac Brûlé, 46.0885°N, 74.2789°W, 370 m: 1 ♂, 4 Jul 1993, day sweep in mixed forest, J.-F. Landry, specimen # CNCLEP00076570 (CNC); Lac Brûlé, 46.0909°N, 74.2756°W, 370 m: 4 ♂, 2 ♀, 7 Jul 1993, day sweeping in shaded spruce-birch forest swamp, J.-F. Landry, specimens # CNCLEP00076571–00076576 (CNC); Lac Brûlé, 46.0885°N, 74.2789°W, 370 m: 1 ♂, 9 Jul 1993, at mercury light in mixed forest, J.-F. Landry, specimens # CNCLEP00076577 (CNC); Lac Brûlé, 46.0885°N, 74.2789°W, 370 m: 1 ♂, 1 Jul 1996, sweeping ferns at forest edge in afternoon, J.-F. Landry, specimens # CNCLEP00076577 (CNC); Lac Brûlé, 46.0885°N, 74.2789°W, 370 m: 1 ♂, 30 Jun 1997; 1 ♂, 1 Jul 1997, day sweep in mixed forest, J.-F. Landry, specimen # CNCLEP00076578 (CNC); Lac Brûlé, 46.0812°N, 74.2833°W, 370 m: 2 ♀, 1 Jul 1997, sweep in forest trail ca 18:00H, sunny, J.-F. Landry, specimens # CNCLEP00076580–00076581 (CNC); Lac Brûlé, 46.0919°N, 74.2756°W, 370 m: 13 ♂, 5 ♀, 2 Jul 1997, in swampy wood perched on vegetation 11:30H–12:30H overcast just before thunderstorm, J.-F. Landry, specimens # CNCLEP00076582–00076599 (CNC); Lac Brûlé, 46.0921°N, 74.2756°W, 370 m: 1 ♂, 1 ♀, 2 Jul 2000, afternoon sweeping liverworts and low vegetation in forest swamp, specimens # CNCLEP00067787–00067788, CNC slide MIC5756, DNA barcoded (CNC); 14 ♂, 5 ♀, 8 Jul 2002, day sweeping shaded liverworts near boggy marsh, specimens # CNCLEP00007712–00007720, 00068787–00068788, CNC slides MIC5753, MIC5755, MIC5757, MIC5758, MIC5760, MIC5762, MIC5763, 9 DNA barcoded (CNC); 6 ♂, 29 Jun 2003, in mixed forest swamp day-sweeping herbaceous and shrub vegetation, J.-F. Landry, specimens # CNCLEP00002816–00002821, CNC slide MIC5761, DNA barcoded (CNC); Lac Brûlé, 46.0881°N, 74.2788°W, 370 m: 1 ♂, 1 ♀, 4 Jul 2004, day sweep in forest swamp with liverwort, J.-F. Landry, specimens # CNCLEP00006682–00006683, CNC slide MIC5754, 1 DNA barcoded (CNC). Gatineau Park, Ramsey Lake, Hopkin’s Hole, 45.6025°N, 76.1079°W, 245 m: 12 ♂, 3 ♀, 11 Jun 1991, afternoon sweeping in forest swamp, J.-F. Landry, specimens # CNCLEP00076600–00076614 (CNC). Gatineau, Masham Township, 45.68°N, 76.05°W: 1 ♂, 26 Jun 1974; 1 ♀, 30 Jun 1974, D.M. Wood, specimens # CNCLEP00077284–00077285 (CNC). UNITED STATES: GEORGIA: Rabun Co: Chattahochee National Forest, Tate Br. Campground: 1 ♀, 16–17 May 1970, O. S. Flint, Jr. (USNM). KENTUCKY: Powell Co: 1 ♂, 23 Nov 1909, 1 ♂, 25 May 1924 (USNM). MAINE: Aroostook Co: Round Mountain: 1 ♂, 20 Jul 1956. Piscataquis Co: Greenville: 1 ♂, 9 Jul (USNM). Franklin Co: West Farmington: 1 ♂, 29 Jun 1966, A. E. Brower (USNM). Hancock Co: Acadia National Park, Mt. Desert Island: 1 ♂, 30 Jun 1933, (USNM). Penobscot Co: Passadumkeag: 1 ♀, 25 Jun 1938 (USNM). Piscataquis Co: Baxter State Park, Mt. Katahdin, Hunt Trail, 2400 feet: 1 ♂, 17 Jul 1948, bushes by brook, A. E. Brower, slides 16388, wing USNM 29861 (USNM). Sagadahoc Co: Woolwich: 1 ♀, 29 Jun 1965, A. E. Brower, slide USNM 33917 (USNM). MARYLAND: Montgomery Co: Cabin John: 1 ♂, 30 Apr 1921, A. Busck (USNM). MICHIGAN: Keweenaw Co: Isle Royale National Park: 2 ♂, 10 Jul 1957, R. W. Hodges (USNM). Emmet Co: Wilderness State Park, 45.7119°N, 84.9402°W, 180 m: 6 ♂, 30 Jun 1992, 17:00–18:00 hrs sweeping liverworts on banks of shaded stream in oak-pine forest with thuja, J.-F. and B. Landry, specimens # CNCLEP00068781–00068786, CNC slides MIC5752, MIC5764, DNA barcoded (CNC). NEW HAMPSHIRE: Rockingham Co: Hampton: 1 ♂, 6–11–1904, S.A. Shaw (USNM). NEW JERSEY: Essex Co: Essex Co. Park: 1 ♂, 3 Jun 1900, W. D. Kearfott (USNM). Essex Co: 1 ♂, 3 Jun; 7 ♂, 3 ♀, 8 Jun 1907, W. D. Kearfott, slides USNM 18409, 91794, 91795 (USNM). NEW YORK: Essex Co: [Keene]: Table Top Mountain, 3500 feet: 2 ♂, 1 ♀, 21 Jul 1940 (USNM). NORTH CAROLINA: Swain Co: Great Smoky Mountains National Park: Oconaluftee River at Towstring Road: 1 ♂, 11 May 1970, SEM slide USNM 17565 (USNM). Smokemont Campground and nearby: 2 ♀, 11–14 May 1970, slide USNM 33920, head slide 16614 (USNM). Whitewater River at rt. 171: 1 ♂, 18 May 1970, O. S. Flint, Jr. (USNM). PENNSYLVANIA: Dauphin Co: Inglenook: 1 ♀, 30 May 1911 (USNM). SOUTH CAROLINA: Pickens Co: Clemson, Wildcat Creek: 1 ♂, 25 Apr 1968, P. Carlson, J. Morse (USNM). TENNESSEE: Sevier Co: University of Tennessee Field Station, 35.739°N, 83.4235°W, 503 m: 1 ♀, 22 May 2005, afternoon sweeping vegetation along forest creek, J.-F. Landry, specimen # CNCLEP00016403, CNC slide MIC5759, DNA barcoded (CNC). VIRGINIA: Falls Church: 1 ?, 1908, A. Busck, wing slide USNM 91787 (USNM). WEST VIRGINIA: Pendleton Co., Smoke Hole State Park, Briggs Run: 3 ♂, 2 ♀, 28 May 1977, [sweeping low vegetation 8–11 AM], D. & M. Davis, slide USNM 20690 (USNM).

#### Distribution

(Fig. 32).*Epimartyria auricrinella* occurs widely over eastern North America, in Canada from Nova Scotia to Ontario, and in the U.S. from Maine to Michigan and south to Tennessee and Georgia.

#### Remarks on larval morphology.

*Chaetotaxy*:Because the larvae of Micropterigidaelacksome thoracic and abdominal setae present in higher Lepidoptera, determining the homology of those setae present is subject to uncertainty. Various assumptions have been made as to which setae are present, based in part on their position to longitudinal muscle groups or to various body ridges (Hashimoto 2001, 2006, Gibbs 2010), as well as to the generally accepted chaetotaxy of glossatan Lepidoptera (Hinton 1946, Stehr 1987) which was followed by Davis (1987). Greatest uncertainty persists with the prothoracic chaetotaxy, where the number and relative development of setae can vary between different genera of Micropterigidae. Hashimoto (2001) concluded that the XD (of the prothorax) and the SD groups are absent in Micropterigidae, with the possibility that the two most dorsal of the four pairs of peg-like microsetae along the anterior margin of the prothorax in *Epimartyria* could be vestiges of one or more of these groups. The more ventral of the two microsetae along the anterior margin of the prothorax are believed to represent MV1 and MV2 present in most Lepidoptera, but homology of the dorsal pair is questionable. Because microsetae in this region are not known to occur on the prothorax of other Lepidoptera (Hinton 1946), we have considered the dorsal pair to be homologous to XD1 and XD2 as suggested by Hashimoto (2001). Hinton (1946) briefly discussed the possibility that the XD group in higher Lepidoptera might be homologous to the microsetae of other body segments, but he argued that long tactile setae along the front margin of the prothorax represented instead a special setal group essential for protecting larvae, especially in those species with prognathous, retractable heads. In Micropterigidae it appears as if this protection has been compensated by several of the prothoracic tactile setae being directed strongly forward (Fig. 33). It may also be possible that several proprioreceptor (microscopic) setae homologous to those of higher Lepidoptera do not occur in Micropterigidae, and that all or most of the relatively stout microsetae present may represent greatly reduced tactile setae. Such reductions have occurred with the abdominal L2 and SV2 setae of *Epimartyria* (Fig. 33), D1 and D2 of *Austromartyria* (Gibbs 2010), and D2 of *Agrionympha* (Gibbs and Kristensen 2011). All microsetae of *Epimartyria auricrinella* are similar to the prothoracic microsetae in being relatively stout and greatly reduced it length (Fig. 33). Consequently, in this study we have largely followed the protothoracic chaetotaxy proposed by Hashimoto (2006) for the closely related genera *Paramartyria* and *Neomicropteryx*. The number and distribution of the prothoracic microsetae have not been well studied or illustrated in most genera of Micropterigidae. Better resolution of the prothoracic setal homology might become possible as larvae of more genera are discovered and studied.

*Prolegs*: The larval prolegs of Micropterigidae, which occur on abdominal segments 1–8 and 10, differ in their morphology from those of all other Lepidoptera where crochet- bearing prolegs are typically present only on segments 3–6 and 10. Hinton (1958) also reported muscles to be lacking in micropterigid prolegs, although this probably should be examined further in some genera such as *Micropterix* where the prolegs appear more developed and with more melanized, acute, clawlike apices (Figs 66–68). The anal prolegs of *Micropterix* are also distinct in forming a relatively broad, trilobed sucker (Fig. 66; Zeller-Lukashort et al. 2007).

*Integumental specializations*: Larvae of Micropterigidae often occur close to the ground in habitats more likely to be subjected to periodic flooding and drying. As an adaptation to such conditions, the larvae have developed an unusual cuticular morphology in the form of a physical gill, or plastron (Thorpe 1950, Davis 1987), which provides extensive air–water interface for gaseous exchange. The aquatic larvae of several species of Crambidae have also developed special gills and plastron cuticles for breathing underwater (Wichard et al. 2002).

An extensive plastron area has been observed in *Epimartyria* (Davis 1987), and similar cuticular structures with various modifications appear in other genera of Micropterigidae examined. The plastron in *Epimartyria auricrinella* extends as a broad band laterally around the body between the level of the lateral (L1) and subventral (SV1) setae and then dorsally over the posterior margin of the prothorax (Fig. 33). The abdominal spiracles are located near the dorsal margin of the band at the extreme anterior edge of the segment (Fig. 52). The surface of the integument within this zone is densely covered with minute, irregularly shaped micropapillae (Figs 52, 55). Radiating out between adjacent micropapillae are dense series of even smaller ridges, aligned ~ 0.4–1.0 µm apart. Each ridge bears a single row of elongate, erect, knobby microtubercules ~ 0.2–0.4 µm in diameter and ~ 0.8–1.2 µm in length (Fig. 56). These minute structures are believed to help form an air film around that portion of the body (when submerged) that excludes water under normal hydrostatic pressure. Minute openings in the epicuticle are visible between the ridges (Fig. 57). These lead internally into an unusually complex, multichambered exocuticle reported by Kristensen (1990, 1998) in the larvae of *Sabatinca* and *Micropterix*. The basal layer of the exocuticle was found in these genera to possess small pores in each chamber which opened into a fluid- filled space between the exo- and endocuticle. Kristensen hypothesized the function of these unique cuticular specializations may be to assist in maintaining a water balance for larvae in a habitat subjected to periodic drying. An extremely thin, extracuticular pellicle covers much of the dorsal, lateral, and part of the ventral larval trunk to which small particles may adhere. The function and origin of the pellicle remain unknown. Immediately beneath the abdominal pellicle of *Epimartyria auricrinella* the exocuticle is divided into a series of honeycombed chambers (Fig. 58) resembling the condition Kristensen discovered in the larvae of *Sabatinca* and *Micropterix*.

The multidentate, scale-like cuticular outgrowths (Figs 41–42) covering the intersegmental membrane between the head and prothorax of *Epimartyria auricrinella* may further assist in a respiratory function. These structures superficially resemble the plastron scales present in certain Coleoptera (Hinton 1969, 1976). The spiracles in *Epimartyria* (Figs 53–54) are also modified to prevent water entry. Each spiracle is raised into a small dome with finely divided, fimbriated walls. The spiracles in later instars of *Neomicropteryx* larvae are also conical with fimbriated walls, but the spiracular walls of the first instar are completely fused (i.e., solid) (Hashimoto 2006) as they are in later instars of *Sabatinca* and *Micropterix* (early instars not examined). Spiracles of the first instar of *Epimartyria* have not been examined but may be similar to those of *Neomicropteryx*.

The larval plastron of *Neomicropteryx nipponensis* Issiki is similar to that of *Epimartyria auricrinella* in possessing a dense zone of minute, irregularly shaped micropapillae interconnected by dense radiations of smaller ridges bearing rows of knobby microtubercules (Figs 60–62). Scutate outgrowths also arise from the intersegmental head-prothoracic membrane (Fig. 63) as in *Epimartyria*. It is likely that larvae of all members of the northern hemisphere group of micropterigid genera proposed by Kobayashi et al. (2000) and Gibbs et al. (2010) have developed similar plastron specializations. In contrast, the external surface of the exocuticle of *Micropterix* (Figs 70–72) possesses a more extensive, regular arrangement of micropapillae, each ~ 10–20 µm in diameter, with ~ 6- 8 relatively stouter, often bifurcate, arm-like ridges radiating from a central disk. The ridges in *Micropterix* do not continue with those of adjacent ridges, but the extremities of each ridge are densely covered with microtubercules. Minute openings of variable size are present in the exocuticle of *Micropterix* (Figs 71–72),similar to those observed in *Epimartyria* and *Neomicropteryx*.

**Figure 12a.**
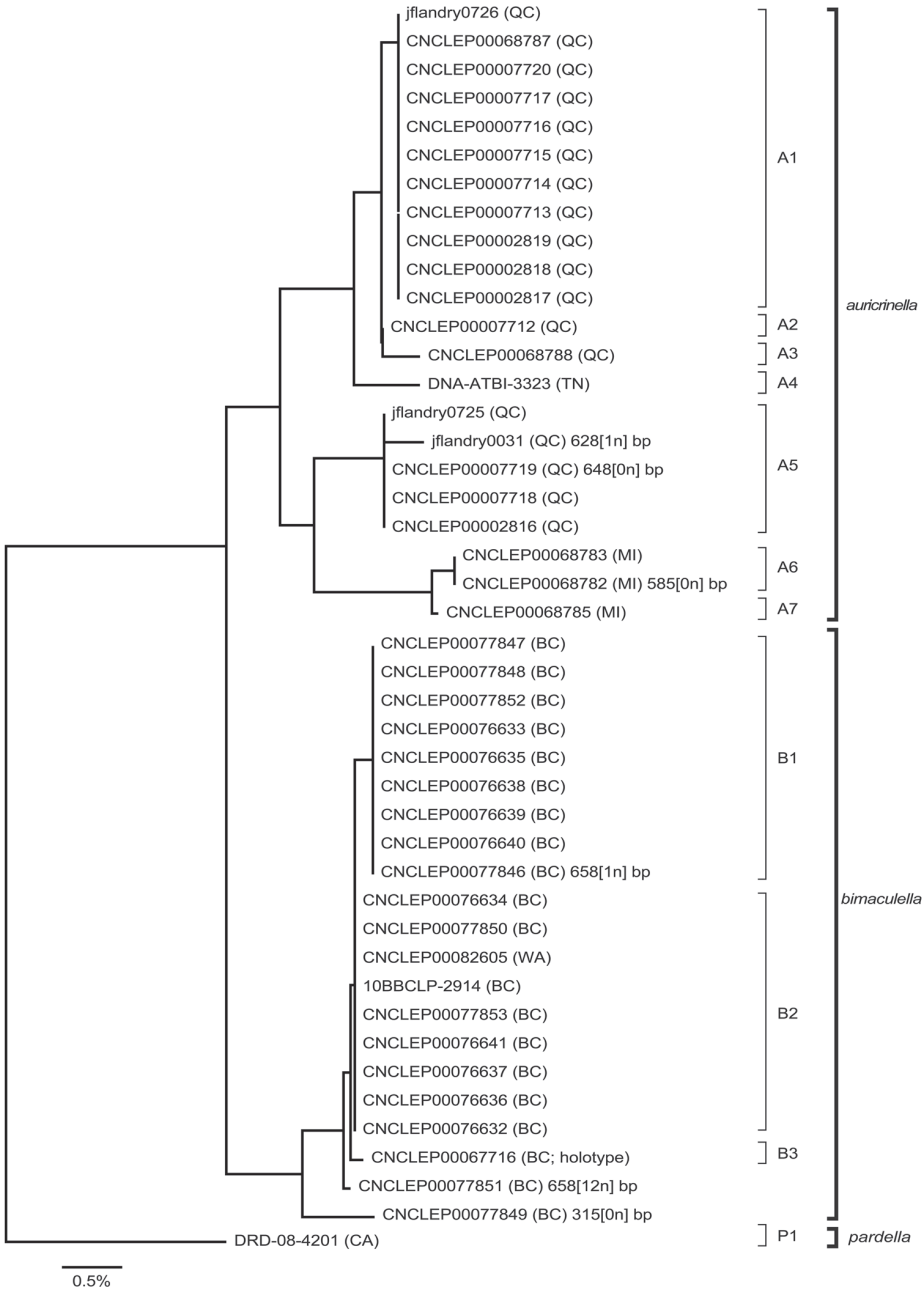
Neighbour-joining tree of genetic distances (K2P model) for cytochrome c oxidase I (COI) in species of *Epimartyria* (total = 44 specimens). End-branch labels are specimen Sample IDs followed by the geographic area in parentheses: BC = British Columbia; CA = California; MI = Michigan; QC = Quebec; TN = Tennessee; WA = Washington. Sequence lengths are 658bp unless otherwise indicated (xn in square brackets indicates the number of ambiguous positions). Distinct haplotypes are designated by a capital letter and digit.

**Figure 12b.**
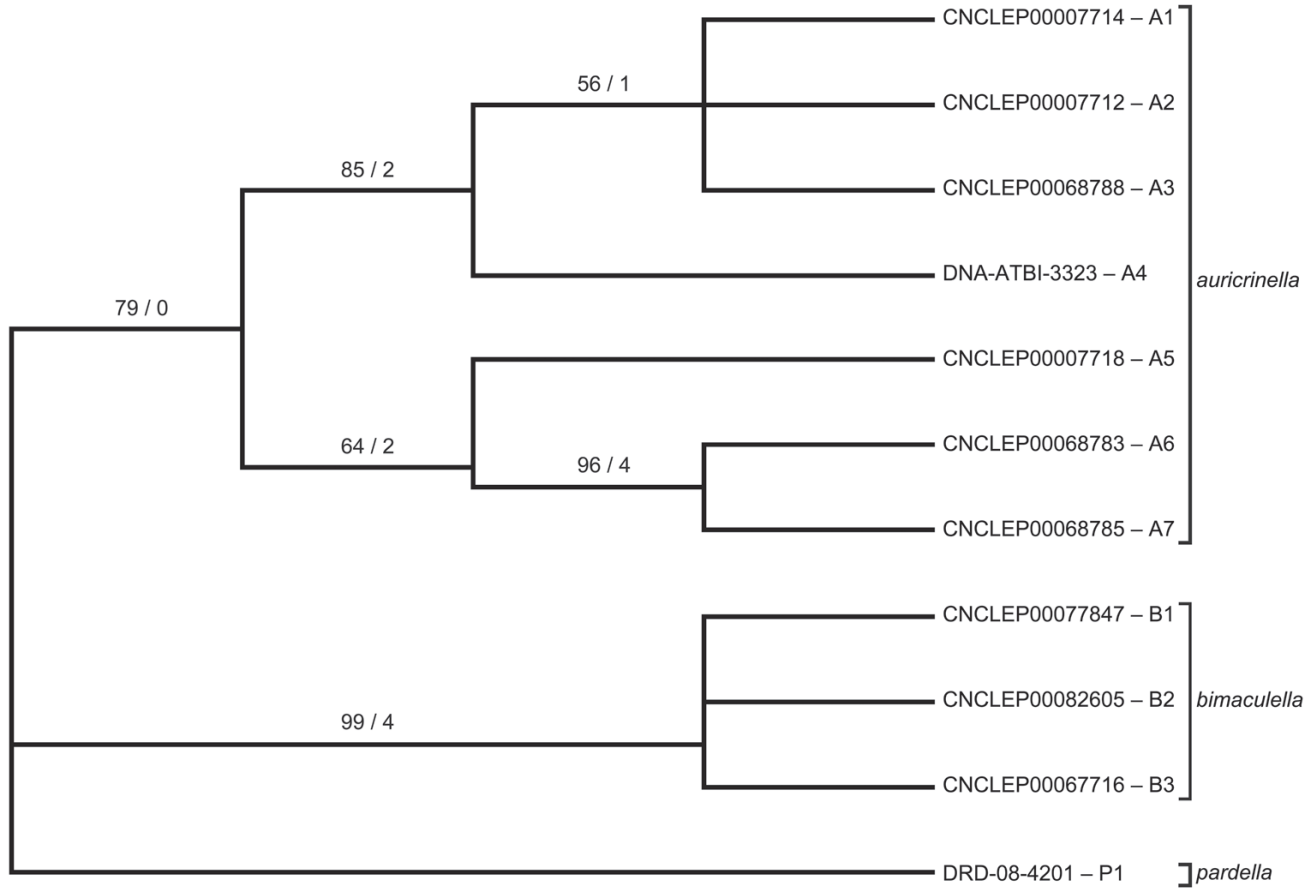
Strict consensus tree of three most parsimonious trees (length = 65, CI = 0.877, RI = 0.857) based on 11 unique DNA barcode haplotypes in species of *Epimartyria*. End-branch alphanumeric labels are specimen SampleIDs with haplotype designations (A1, A2, etc.). Numbers above branches are bootstrap values (1000 replicates) / Bremer support values.

**Figure 12c.**
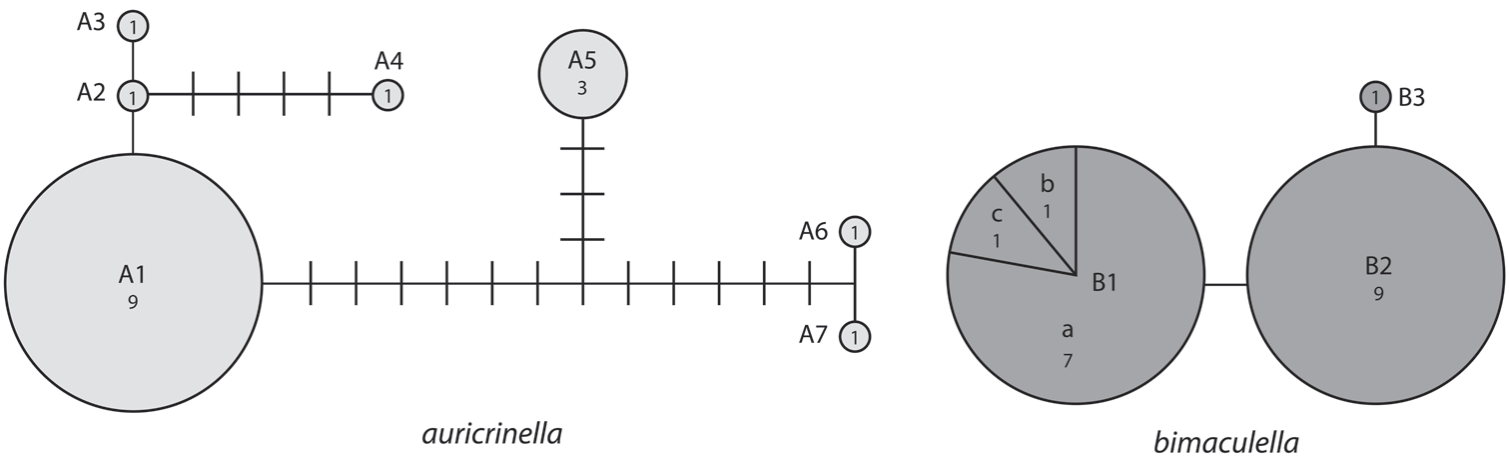
Haplotype network for 10 distinct haplotypes detected in two species of *Epimartyria* (7 for *Epimartyria auricrinella*, 4 for *Epimartyria bimaculella*). Circles are labelled with the haplotype name (capital letter), and the number of specimens per haplotype; lower case letters refer to localities indicated on the distribution map (Fig. 32). The single sequence of *Epimartyria pardella*, which separated out, is not shown.

**Figures 13–17.**
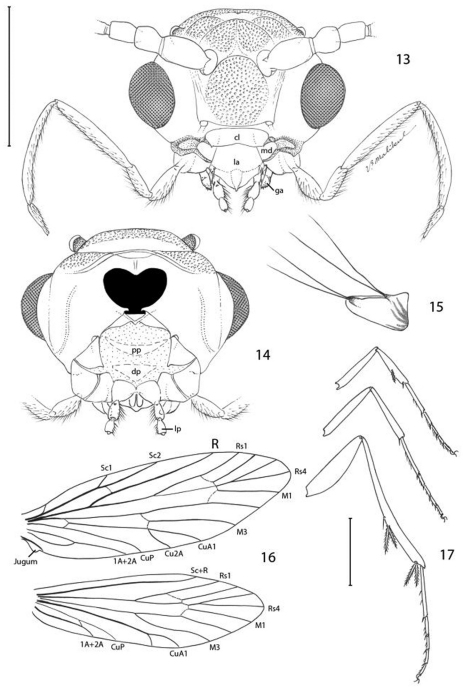
*Epimartyria pardella*, Adult morphology **13** Head (cl: clypeus; ga: galea; la: labrum; md: mandible) (0.5 mm) **14** Head, ventral view ( pp: proximal prelabium; dp: distal prelabium; lp: labial palpus) **15** right mandible **16** Wing venation, USNM slide 16613. **17** Legs (1.0 mm). (Scale lengths in parentheses).

### Epimartyria bimaculella sp. n.

urn:lsid:zoobank.org:act:6A40EFD1-BC6D-4DF3-AB1C-D4031870E61D

http://species-id.net/wiki/Epimartyria_bimaculella

Figs 2 6–7 11 32 81–87

#### Diagnosis.

Adultsof *Epimartyria bimaculella* most resemble those of *Epimartyria pardella* in possessing dark fuscous forewings marked by pale golden spots. A total of two yellowish spots occur in *bimaculella*, with only a single large costal spot present beyond the middle of the forewing. Four spots are present on the forewing of *pardella*, with two of these located across the distal third of the wing on the costal and dorsal margins respectively.

#### Adult

(Figs 2, 6–7). *Head*: Vestiture similar to *Epimartyria auricrinella*, light orange brown. Antenna with vestiture of scape and pedicel concolorous with head; scales of flagellum mostly pale golden brown, becoming darker, more fuscous over distal third. Labial palpus cream.

*Thorax*: Dark fuscous with coppery to purplish luster. Tegula concolorous with head. Forewing mostly dark fuscous with coppery to purplish luster dorsally, marked with two pale yellowish spots; the largest, irregularly oval to rectangular spot extends from the costa approximately halfway across the distal third of wing; a second smaller, more slender spot extends diagonally from about midway along dorsal margin to midway on discal cell; a slight suffusion of pale yellowish scales may be sometimes evident at the base of the forewing, but only seldom does this occur; forewing less iridescent ventrally; fringe pale yellow along termen, fuscous along dorsal margin. Forewing length: 4.6–5.3 mm. Hindwing mostly gray, becoming darker and slightly iridescent toward apex; fringe gray. Legs medium to dark brown dorsally with a slight purplish luster, light brown to cream ventrally; epiphysis absent.

*Abdomen*: Piliform scales dark brown dorsally, paler brown ventrally.

*Male genitalia* (Figs 81–85): Tergum X similar to *Epimartyria auricrinella*, broadly bilobed. Caudal apex of sternum X deeply divided, with apex of lobes acute, only slightly curved; a pair of short, lateral lobes present near base. Valva moderately long, ventral length ~ half the maximum length of segment IX; apex subacute and bearing a short, slender, recurved spine similar to *Epimartyria auricrinella*; a short but broader and more triangular, rounded process arising midway from mesal surface; elongate basal process ~ 4/5 the length of valva; distal margin of valva variable within populations from slightly convex to ~ straight. Dorsal branch of phallus cylindrical and smooth.

*Female genitalia* (Figs 86–87): As described for genus. Caudal end of genital sclerite moderately furcate as in *Epimartyria auricrinella*; length of furcations ~ 0.3 that of moderately long, undivided base.

#### Larva and pupa.

Unknown.

#### Biology

(Figs 6–7, 11). At the type locality, specimens were captured by sweeping low lying vegetation or during diurnal flight along a shaded seepage in a Douglas Fir–Western Red Cedar forest where leafy liverworts grew. Adults were also observed perching on lower parts of plants such as Salmonberry (*Rubus spectabilis* Pursh) no more than approximately 25 metres from the liverwort habitat (D.G. Holden, pers. comm.). In different parts of the range, specimens were collected from late April to mid August, with most records in June. Late records (July and August) are from higher elevations.

#### Holotype.

♂, CANADA: BRITISH COLUMBIA: Belcarra, 49°17'59.11"N, 122°55'30.88"W, Alt. 25 m., 8 Jun 2008, visual sweep, Dave G. Holden, specimen # CNCLEP00067716, CNC slide MIC5768, Barcode of Life Project, leg removed, DNA extracted, digital image captured, (CNC).

#### Paratypes.

CANADA: BRITISH COLUMBIA: Belcarra Park, 49.3107°N, 122.9263°W, alt. 13 m: 2 ♂, 24 May 2009, day sweep, Dave G. Holden, specimen # CNCLEP00076632–00076633 [both DNA barcoded] (CNC); 13 ♂, 1 ♀, 1 Jun 2009, day sweep, Dave G. Holden, specimens # CNCLEP00076634–00076639, 00077846–00077853, CNC slides MIC5765, MIC5767, MIC5766, MIC5570, MIC5571 [all DNA barcoded] (CNC); 2 ♂, 2 Jun 2009, day sweep, Dave G. Holden, specimens # CNCLEP00076640–00076641 [both DNA barcoded] (CNC). Maple Ridge, Univ. of British Columbia Research Forest, 49.277679°N, 122.553870°W, 259 m: 1 ♂, 1 Jun 2011, visual sweep, Dave G.Holden (CNC). Fraser Mills: 6 ♂, 7 ♀, 11 June 1921, L. E. Marmont, SEM slide USNM 18431, slides USNM 33919, 98001, 98002, 98004; 27 ♂, 7 ♀, 15 Jun 1922, E. H. Blackmore collector, slides USNM 17503, 18410–18411, 34282, 91785, 97991, 98000, 98003, 98005, 98009–98017 (BMNH, CNC, USNM). Squamish, Diamond Head Trail: 1 ♂, 12 Aug 1963; 2 ♂, 14 Aug 1963, W.R.M. Mason, specimens # CNCLEP00077292–00077294, CNC slide MIC1826 (CNC). Mt Seymour, 49.337368°N, 122.957695°W, 292 m: 1 ♂, 21 Jun 2011, visual sweep, Dave G.Holden (CNC). Glacier National Park, Loop Trail, 1140 m, 51.254°N, 117.538°W, 1 ♀, 16 Jul 2010, malaise trap, specimen #10BBCLP-2914 [DNA barcoded], CNC slide MIC5769 (BIO). UNITED STATES: Washington: Clallam Co: Olympic National Park, sweeping on Soleduck Trail to Deer Lake, 1000 m: 3 ♂, 15 Jul 1998, D. R. Davis, slide USNM 34302 (USNM). Olympic Peninsula, Port Angeles, 245m, 48,07924°N, 123,42990°W ± 50m: 3 ♂ , 1 ♀, 20 Jun 2010; 1 ♂, 20.6.2010, 15:45h, Hausenblas and Zeller-Lukashort (ONPS). Olympic Peninsula, Sol Duc Hot Springs Rd, 390m, 48.06385°N, 123.99565°W ± 50m: 1 ♂, 21 Jun 2010, 13:00h, Hausenblas and Zeller-Lukashort, slide AP-Nr 42/2010 Christof Zeller (ONPS). Olympic Peninsula, Kalaloch, 10m 47.61131°N, 124.37588°W ± 50m: 1 ♂, 23 Jun 2010, 17:00h, Hausenblas and Zeller-Lukashort (ONPS). Olympic Peninsula, Hoh Rainforest Rd, 130m 47.81641°N, 124.05161°W ± 50m: 21 ♂ , 3 ♀, 22 Jun 2010, 16:15h, Hausenblas and Zeller-Lukashort (CZC). Grays Harbor Co: Elma: 46.9738°N, 123.2945°W, yel st trp, 1 ♀, 27 Jun 2011, G. Kohler, WSDA 978–1008A (WSCAD). King Co: Asahel Curtis picnic area: 47.3951°N, 121.4677°W, 1 ♂, 27 Jul 2011, hand col, C. Looney, WSDA W666–1129A, B (WSCAD, USNM). Stevens Pass, Hwy 2, 14.5 km E Skykomish, 645 m., 47.7143°N, 121.1722°W: 1 ♂, 8 Jul 2010, afternoon sweep, J.-F. Landry and D.G. Holden, specimen #CNCLEP00082605, CNC slide MIC5739 [DNA barcoded] (CNC). Mason Co: Skokomish River Rd: 47.3019°N, 123.1858°W, 4 ♂ , 2 ♀, 17 Jun 2011, hand col, C. Looney, WSDA W666–1131A-E (WSCAD, USNM). Pierce Co: Fort Lewis: 1 ♂, 29 May 1951, R. Schuster, Essig Museum slide 0152, (UCB). Snohomish Co: East Arlington Co. Park: 1 ♂, 29 Apr 1979, L. Massell, e. *Lepidozia* liverwort, slide USNM 98006 (USNM). 6 mi. E of Verlot: 1 ♂, collected 26 Mar 1979, emerged 2 May 1979, reared from liverwort, *“Jungermannia obovata”* L. Russell (USNM). Thurston Co: Evergreen State College, 47.0791°N, 122.9750°W, 2 ♂, 25 Jun 2011, hand col, C. Looney & E. Lagasa, WSDA 666–1130A, B (WSCAD, USNM).

Additional specimen examined, excluded from type material: CANADA: BRITISH COLUMBIA: Glacier National Park, Loop Trail, 1140 m, 51.254°N, 117.538°W, 1 ♀, 16 Jul 2010, malaise trap, specimen #10BBCLP-2914 [DNA barcoded] (BIO).

#### Distribution

**(**Fig. 32). *Epimartyria bimaculella* is known from northwestern Washington and southern British Columbia. Most British Columbia records are from the southwesternmost corner in the periphery of the Vancouver area, reflecting a more intense collecting effort in that region. One record from the Rocky Mountains of Glacier National Park, BC suggests a significantly broader distribution.

#### Etymology.

The species nameis derived from the Latin *bi*; (two, double) and *maculella* (little spot) in reference to the two, small, pale yellowish spots present on the forewings.

**Figures 18–23.**
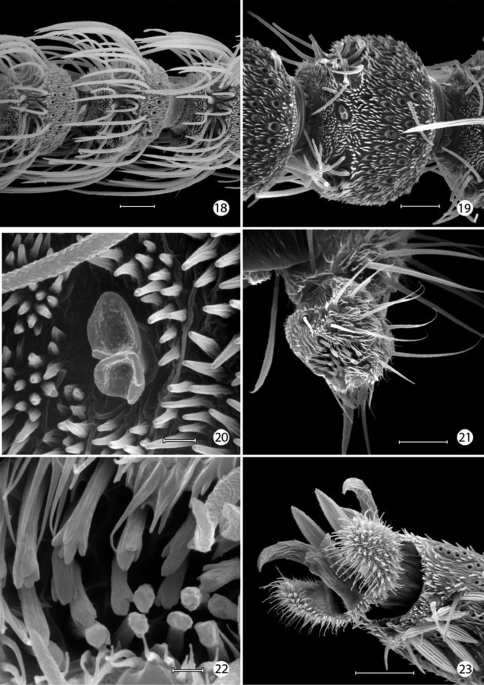
*Epimartyria auricrinella*, Adultmorphology **18** Flagellomeres with ascoid sensilla (20 µm) **19** Flagellomere with multiporus sensillum placodeum (20 µm) **20** Detail of multiporus sensillum placodeum in Fig. 19 (2 µm) **21** Apical segment of labial palpus with distal organ vom Rath (20 µm). **22** Sensilla of organ vom Rath (2 µm) **23** Mesothorcic pretarsus (20 µm). (Scale lengths in parentheses).

**Figures 24–27. F9:**
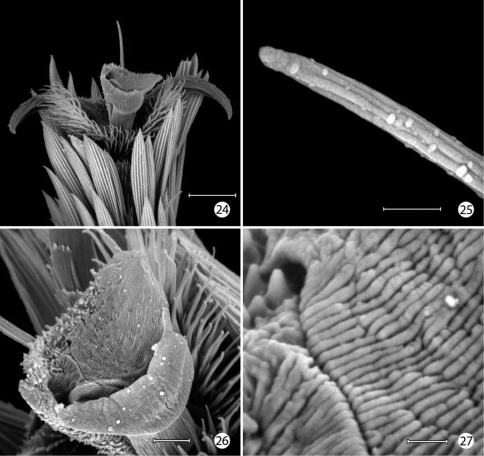
*Epimartyria auricrinella*, Adultmorphology **24** Mesothorcic pretarsus (20 µm) **25** Detail of pseudempodium of pretarsus (2 µm) **26** Arolium (5 µm) **27** Detail of surface of arolium (1 µm). (Scale lengths in parentheses).

**Figures 28–31. F10:**
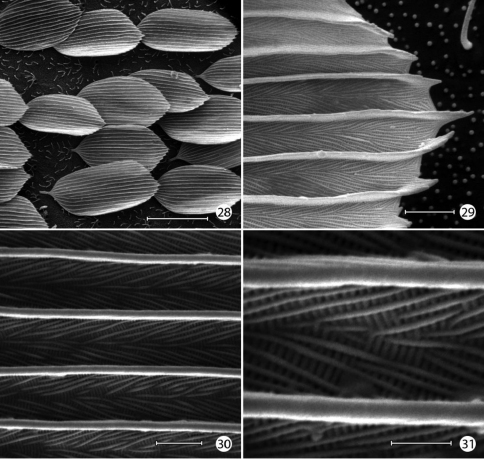
*Epimartyria auricrinella*, Forewing scale structure **28** Dorsal forewing scales from discal cell (40 µm) **29** Apical margin of scale in Fig. 28 (2 µm) **30** detail of Fig. 29 (2 µm) **31** Detail of Fig. 30 (1 µm). (Scale lengths in parentheses).

**Figure 32.**
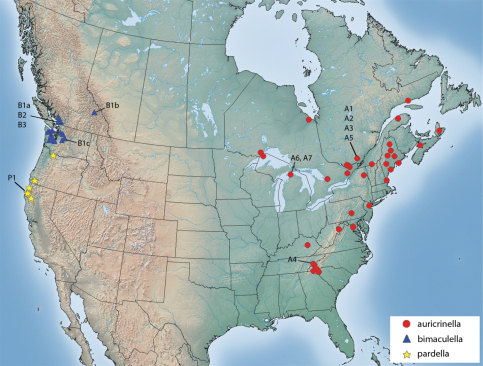
Distribution of *Epimartyria* species. Alphanumeric designations refer to haplotypes shown in the haplotype network of Fig. 12c.

**Figures 33–39.**
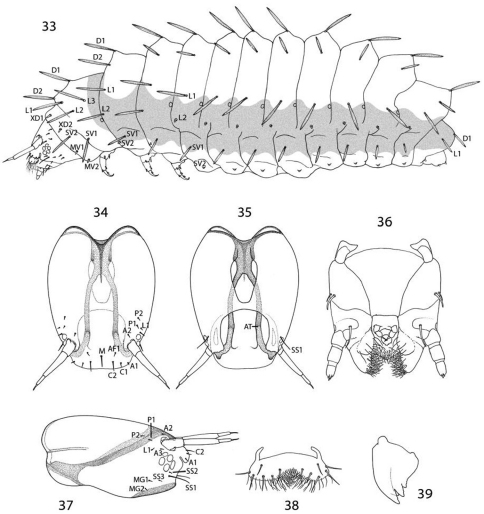
*Epimartyria auricrinella*, larval morphology **33** Chaetotaxy; shaded area indicates extent of epidermal plastron**34** Head, dorsal view(M: medial seta) **35** Head, ventral view (AT: anterior arm of tentorium) **36** Ventralview of maxilla and labrum **37** Head, lateral view **38** Labrum, dorsal view **39** Mandible.

**Figures 40–45. F13:**
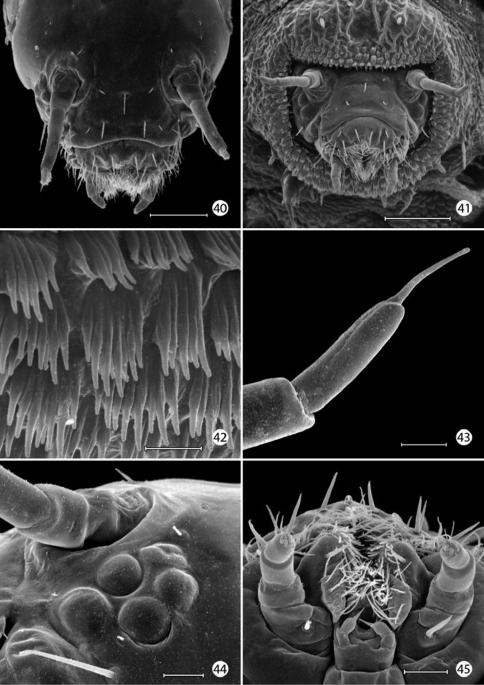
*Epimartyria auricrinella*, larval morphology **40** Head, dorsal view (100 µm) **41** head, anterior view (100 µm) **42** Scutate cuticular outgrowths from head-prothoracic fold (of Fig. 41) (10 µm) **43** Apex of antenna (10 µm). **44** Stemmata, 5 total (25 µm) **45** Ventral view of maxilla and labium (20 µm). (Scale lengths in parentheses).

**Figures 46–51. F14:**
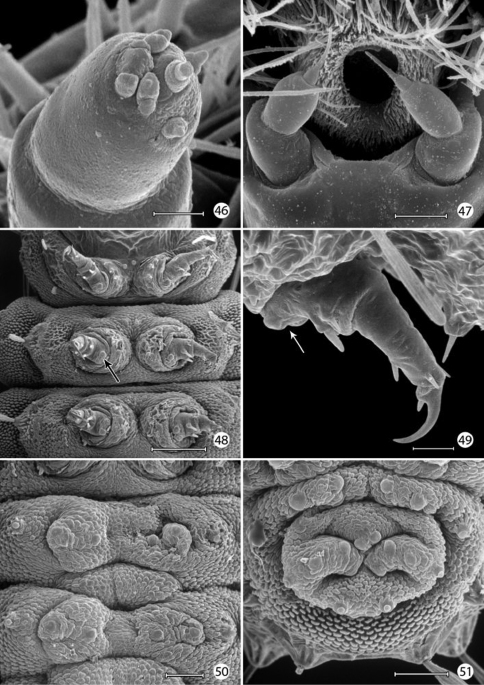
*Epimartyria auricrinella*, larval morphology **46** Apical sensilla of maxillary palpus (5 µm) **47** Labial palpi and opening of labial salivary gland (100 µm) **48** Thoracic legs (arrow: tactile vesicle of coxa) (200 µm) **49** Mesothoracic leg (arrow: tactile vesicle of coxa) (25 µm) **50** Abdominal prolegs, segments 1–2 (100 µm) **51** Anal prolegs (100 µm). (Scale lengths in parentheses).

### Epimartyria pardella

(Walsingham)

http://species-id.net/wiki/Epimartyria_pardella

Figs 3 8 13–17 32 88–96.

Micropteryx pardella Walsingham 1880: 83.
Epimartyria pardella (Walsingham) 1898: 161.– Kearfott in Smith 1903: 125.– Dyar 1903: 581.– Meyrick 1912: 6.– McDunnough 1939: 110.– Davis 1983: 5; 1984: 341.– Kristensen 1984b: 97.– Tuskes and Smith 1984: 40.– Nye and Fletcher 1991: 113.– Poole 1996: 716.– Hashimoto 2006: 43.– Powell and Opler 2009: 33.

#### Diagnosis:

Adults of *Epimartyria pardella* most resemble those of *Epimartyria bimaculella* in possessing dark fuscous forewings marked by pale golden spots. Four spots are present on the forewing of *pardella* with two of these located across the distal third of the wing. In contrast, a total of two yellowish spots occur in *bimaculella*, with only a single large costal spot present beyond the middle of the forewing. In the male genitalia, the caudal lobes of sternum X (uncus) are more simple than those of the other members of *Epimartyria* in consisting of more shallow, rounded lobes compared to being curved and more slender in the males of *auricrinella and bimaculella*.

#### Adult

(Figs 3, 8). *Head*: Vestiture light orange brown. Antenna with vestiture of scape and pedicel concolorous with head; scales of flagellum pale golden yellow. Labial palpus pale brown to cream.

*Thorax*: Dark fuscous with coppery to purplish luster. Tegula concolorous with head. Forewing dark fuscous with coppery to purplish luster dorsally, marked with four, pale yellowish spots; the largest, irregularly rectangular and slightly oblique spot extends from costa approximately halfway across the distal third of wing; a smaller, more oval spot opposite costal spot on dorsal margin; an oblique basal spot arising midway along dorsal margin and extending halfway across wing to base of radial vein; a fourth, smallest spot at base of wing; forewing less iridescent ventrally; fringe pale yellow along termen, more gray along dorsal margin. Forewing length: 5.0–5.5 mm. Hindwing mostly gray, becoming darker and slightly iridescent toward apex; fringe gray; fringe light to dark gray. Legs medium to dark brown dorsally, paler brown ventrally and over tarsomeres; epiphysis present, ~ 1/3 the length of foretibia and arising slightly beyond its midlength.

*Abdomen*: Piliform scales light to dark brown.

*Male genitalia* (Figs 88–92): Tergum X with more slender caudal lobes. Caudal apex of sternum X (gnathos) not deeply divided, with short, triangular caudal lobes and without accessory lateral lobes. Valva short, ventral length less than 1/3 the maximum midventral length of segment IX; apex rounded and bearing a short, stout subapical spine; mesal surface smooth, without median process; elongate basal process nearly equal to length of valva. Dorsal branch of phallus more depressed, with subapical margins bearing short, paired spines, gonopore with less thickened radial folds than in previous two species.

*Female genitalia*: (Figs 93–96): As described for genus. Caudal end of genital sclerite deeply furcated; length of furcations exceeding length of short, undivided base.

**Egg.** White; dimensions 0.44 × 0.44 mm. Tuskes and Smith (1984) report the ova are flattened, circular and smooth when first deposited but soon become spherical and covered with numerous minute projections similar to those reported for *Micropterix calthella* (L.) by Lorenz (1961). The eggs were observed to hatch in 21 days at 22°C.

**Larva.** Not examined. The following description has been summarized from Tuskes and Smith (1984): Body length 4.3 to 4.6 mm; width 1.4 mm; height 1.2 mm. The body tapering at both ends with highest and broadest point at abdominal segment 4. Dorsal and lateral surface brown to dark brown, ventral surface light brown.

*Head*. Length 0.5 mm, diameter 0.27 mm. Brown. Antennae prominent, 3-segmented and situated on small tubercles located on dorsal lateral portion of head. Stemmata with 5 facets and located near base of antenna. Labrum simple with a pair of 3-segmented palpi. Mandibles simple and dark brown. Head diameter of first and second instar larvae 0.11 and 0.22 mm, respectively.

*Thorax:* Prothorax distinctly narrower than mesothorax. Prothoracic shield with 10 peg-like setae, 8 on the anterior and lateral border and 2 dorsally. Mesothorax with 8 setae, 6 on dorsal and lateral anterior portion of gray brown pigmented area, and 2 just ventral to this pigmented area. Setae of metathoracic segment similar to those of mesothorax except subdorsal (D2) seta is greatly reduced in size. All thoracic segments have additional small micro-seta just dorsal to each true leg. Thoracic legs brown, with 3 segments (excluding coxa) and simple claw.

*Abdomen*: Abdominal segments Al to A8 (and T2 and T3) with serrated knobs which form a dorsal and lateral ridge; areas between ridges concave. The mid-dorsal area concave with a small dark depression present on posterior of segments T2 to A8. Segments Al to A8 each with one dorsal seta (length 0.18 mm) atop dorsal ridge. Segments Al to A8 with reduced, almost microscopic subdorsal (D2) seta (length 0.04 mm) and prominent lateral seta (length 0.12 mm) on lateral ridge. Dorsal. subdorsal and lateral setae occur in brownish pigmented area which has rough and wrinkled appearance. Dorsal and lateral intersegmental area constricted and may contain series of 8 to 20 microscopic dots. Cuticle ventral to lateral ridge smooth and light brown. Series of brown dots form pattern around protuberance that usually support a small seta. Conical ventral prolegs occur on segments Al to A8, with a small, sclerotized protuberance present on ventral surface of each. Segments A9 and Al0 fused and with enlarged simple sucker. Spiracles posterior and ventral to lateral setae.

#### Larval hosts.

Hepaticophyta: Conocephalaceae: *Conocephalum conicum* (L.) Dumort.; Pelliaceae: *Pellia* species, with the latter host preferred from rearings (Tuskes and Smith 1984).

#### Pupa.

Not examined. Exarate, decticous; white to light brown.

#### Cocoon.

Not examined. Brown, oval in general form, measuring 5.5 × 4.5 mm; primarily of silk with small fragments of vegetation attached.

#### Biology

(Fig. 8). Tuskes and Smith (1984) observed the eggs of *Epimartyria pardella* to be deposited in June on the underside of liverwort thalli singly or in small clusters of up to five eggs. They are white, measuring ~ 0.40 × 0.44 mm, and are flattened, circular, and smooth when first deposited but become spherical within a short time and covered with a series of small projections. First instar larvae ~ 0.75 mm long were reported to emerge in about 21 days (at 22°C). The larvae are rather inactive (in captivity) and are usually found on the underside of the thalli during the day. When disturbed or inactive the head may be withdrawn into the thorax. Pupation occurs within a thin walled, tightly woven brown cocoon close to the ground and attached to vegetation with strands of coarse silk.

Adults begin to emerge in late May with the flight season ranging from late May to mid- July and peak flight activity in June at the Prairie Creek State Redwood Park locality. Tuskes and Smith reported the adults to be relatively inactive, remaining motionless for hours and seldom travelling more than 30 cm. They are known to be diurnal and most active between 0900 and 1930 h. Adult feeding by *Epimartyria pardella* has not been reported, but the adults were observed drinking water by lowering their heads to the moisture. Moths can die in less than two days if deprived of moisture but may survive in captivity from 9 to 18 days when provided with water.

Tuskes and Smith concluded that *Epimartyria pardella* possessed a two year life cycle similar to that proposed for *Epimartyria auricrinella* (Davis 1987). In captivity eggs deposited in June 1981 became adults in June 1983. In the field they frequently collected second instar larvae during the adult flight period.

#### Lectotype.

♂ (present designation), “OREGON: Klamath Co: nr. Redwood Creek, Coast: 26 June 1872, Wlsm. 90591; B.M. Genitalia Slide No. 25352; *Epimartyria* (= *Micropteryx* Wlsm.) *pardella* Wlsm. PARATYPE; Lectotype ♂, *Epimartyria pardella* Wlsm. (BMNH).” The lectotype has been selected from a series of five syntypes collected **“**on the borders of the forest of “redwood” (*Taxodium sempervirens*) near the coast, in southern Oregon, at the beginning of June 1872” (Walsingham 1880).

#### Material examined.

UNITED STATES: CALIFORNIA: Humbolt Co: Arcata: 1 ♂, 11 Jun 1969, slide USNM 16613, wing slide USNM 18441 (USNM). Fern Canyon: 1 ♀, 18 Jun 1977, N. J. Smith, slide DRD 4529 (UCB). Fern Canyon, Prairie Creek State Park: 3 ♂, 19 Jun 1981, Ann & Paul Tuskes; 1 ♂, 20 Jun 1981, P. Tuskes, 3 ♂, Ann & Paul Tuskes (USNM). Kneeland: 69 Prairie Lane: 1 ♂, 17 Jun 2001, 2 ♂, 18 Jun 2001, 1 ♂, 29 Jun 2001, 1 ♂, 30 Jun 2001, R. S. Wielgus, diurnal flight in wet meadow below house, slide USNM 34278 (USNM); 1 ♀, 22 Jun 2003, R. S. Wielgus, slides DRD 4528, 4529 (UCB). Trinity Co: Forest Glen: 2 ♀, 25 May 1973, J. Doyen (UCB). OREGON: Klamath Co: nr. Redwood Creek, Coast: 1 ♂ (lectotype), 26 June 1872, Wlsm. 90591; B.M. Genitalia Slide No. 25352, (BMNH). Multnomah Co: Benson State Park, Multnomah Falls: 2 ♂, C. V. Piper, 1 ♂, wing slide USNM 91788 (USNM).

#### Distribution

(Fig. 32). *Epimartyria pardella* is known from northwestern California and northern Oregon. California localities and the type locality in Oregon are near the coast in redwood forests. The most northern Oregon locality occurs in the Columbia River valley.

#### Remarks.

Information included in this report on the immature stages and life history of *Epimartyria pardella* has been quoted or summarized from the thorough study of this species by Tuskes and Smith (1984) at the Prairie Creek State Redwood Park, California. In addition to possible color differences, two major morphological differences noted in their description of the larva of *Epimartyria pardella* from that observed for *Epimartyria auricrinella* include: (1) 10 versus 11 (in *auricrinella*) peg-like setae on each side of the prothorax, and (2) D2 of metathorax and abdominal segments 1–8 greatly reduced in *pardella* (as reported also in *Austromartyria*, Gibbs 2010, and for the abdomen in *Agrionympha*, Gibbs and Kristensen 2011). Although examples of the larva, pupa, and cocoon of this species were reportedly deposited in the collections of the California Academy of Sciences, San Francisco, CA by Tuskes and Smith (1984), attempts to locate and borrow this material for study were unsuccessful. The skeletomuscular anatomy of the male genitalia of *Epimartyria pardella* was reviewed by Kristensen (1984b).

**Figures 52–57. F15:**
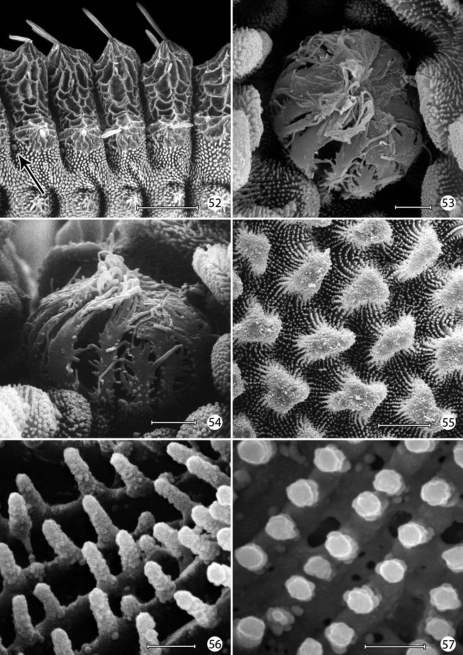
*Epimartyria auricrinella*, larval morphology **52** Abdominal segments 1–4, lateral view, showing sculptured epicuticle of dorsal half and plastron region (shaded area) of lower half (arrow indicates spiracle) (200 µm) **53** Spiracle, apical view (5 µm) **54** Spiracle, lateral view (5 µm) **55** Plastron of lateral surface of abdomen with numerous, irregular micropapillae (10 µm) **56** Detail of fig. 55 showing parallel rows of microtubercules extending between micropapillae (1 µm) **57** View looking down on microtubules in fig. 56 showing cuticular openings between rows (1 µm). (Scale lengths in parentheses).

**Figures 58–59.**
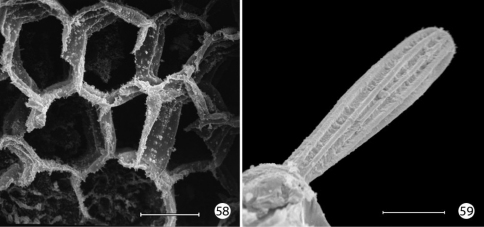
*Epimartyria auricrinella*, larval morphology **58** Honeycombed chambers of abdominal exocuticle with pellicle removed, in dorsal half (dorsal to spiracle) of abdominal segment 4 (10 µm) **59** Abdominal seta D1 showing longitudinal ridges (20 µm). (Scale lengths in parentheses).

**Figures 60–63.**
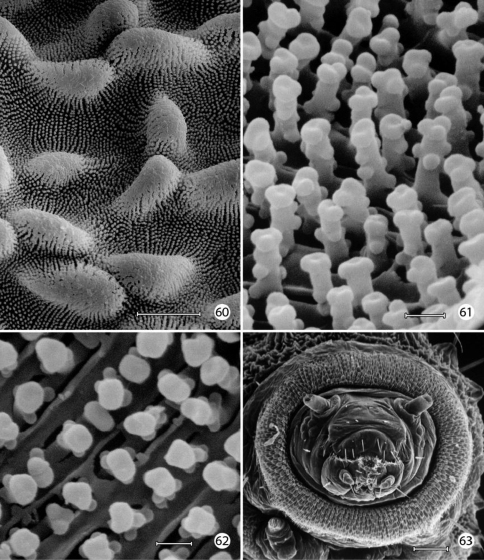
*Neomicropteryx nipponensis*, larval morphology **60** Plastron from lateral surface of abdomen with numerous, irregular micropapillae (10 µm) **61** Detail of Fig. 55 showing parallel rows of microtubercules extending between micropapillae (0.5 µm) **62** View looking down on microtubules in fig. 61 showing cuticular openings between rows (0.5 µm) **63** Head, anterior view (100 µm). (Scale lengths in parentheses).

**Figures 64–69.**
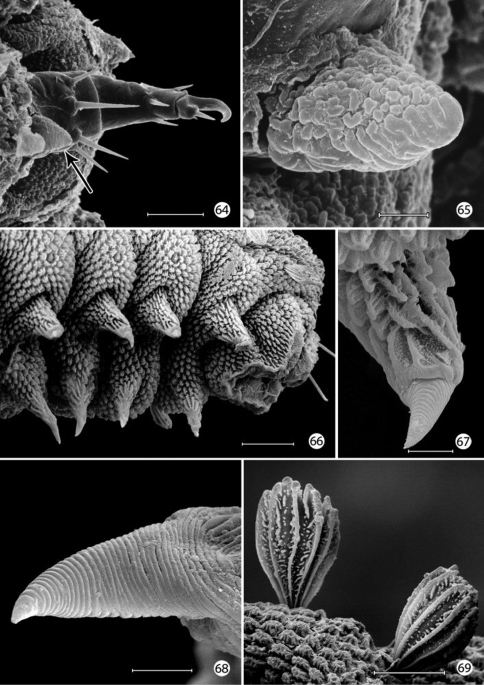
*Micropterix* species (England), larval morphology **64** Prothoracic leg (arrow: tactile vesicle of coxa) (50 µm) **65** Detail of tactile vesicle in fig. 64 (10 µm) **66** Abdominal segments 5–10 showing prolegs 5–8 and sucker-like anal proleg (100 µm) **67** Abdominal proleg (20 µm) **68** Detail of apex of abdominal proleg (10 µm) **69** Dorsal abdominal setae (50 µm). (Scale lengths in parentheses).

**Figures 70–73.**
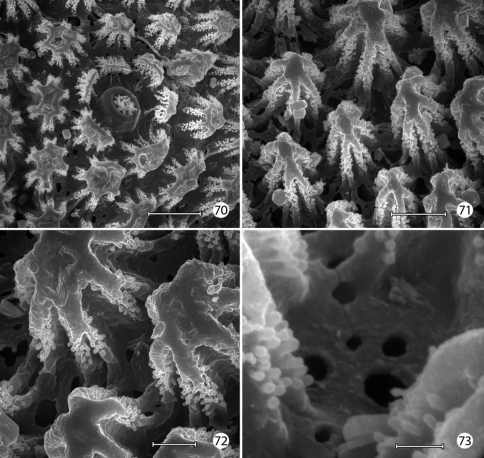
*Micropterix* species (England), larval morphology **70** Lateral plastron surface of abdomen showing micropapillae around broken scale base (20 µm) **71** Detail of plastron Fig. 70 showing cuticular openings between micropapillae (10 µm) **72** Detail of fig. 71 (5 µm) **73** Detail ofcuticular openings in Fig. 71 (2 µm). (Scale lengths in parentheses).

**Figures 74–80.**
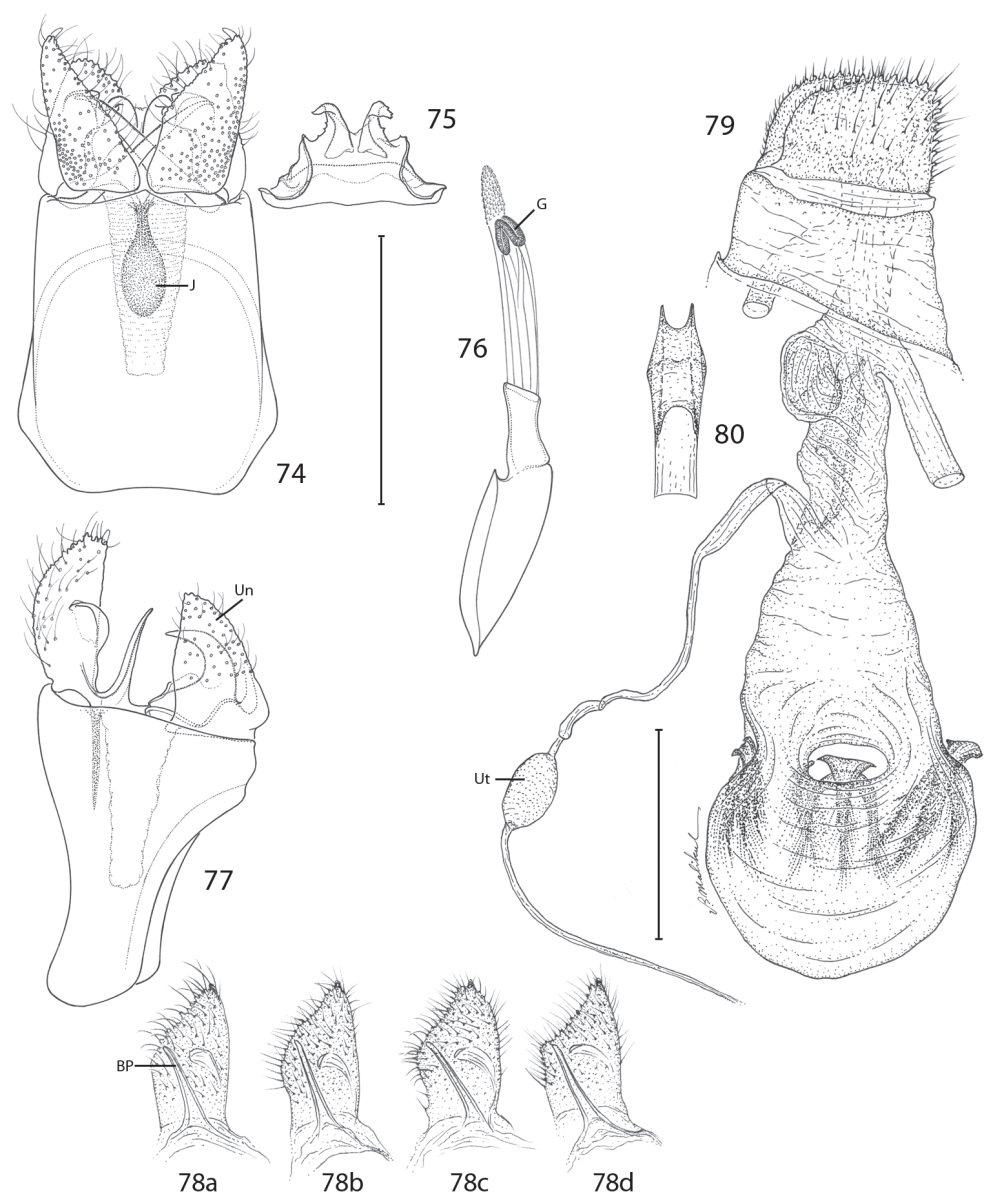
*Epimartyria auricrinella*, Genitalic morphology **74–78** Male, USNM slides 16615, 34372 **74** Genital capsule, ventral view (0.5 mm); J: juxta (medial plate) **75** Sternum X (gnathos) **76** Aedeagus (G: gonopore (phallotreme) **77** Genital capsule, lateral view (Un: uncus, (tergum X) **78a** Valva, lateral view, inner side (BP: basal process), slide USNM, 34372, Ottawa, Ontario **78b** slide MIC5762, Lac Brûlé, Quebec **78c** slide MIC5764, Wilderness State Park, Michigan **78d** slide MIC5761, Lac Brûlé, Quebec **79–80** Female, USNM slide 17501, Mt. Albert, Quebec **79** Oviscape, lateral view (Ut: utriculus) (0.5 mm) **80** Genital sclerite, ventral view. (Scale lengths in parentheses).

**Figures 81–87.**
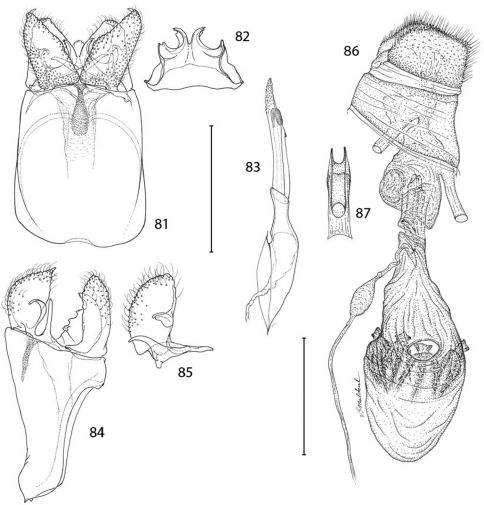
*Epimartyria bimaculella*, Genitalic morphology **81–85** Male, USNM slide 18410, Fraser Mill, British Columbia **81** Genital capsule, ventral view (0.5 mm) **82** Sternum X (gnathos) **83** Aedeagus **84** Genital capsule, lateral view **85** Valva **86–87** Female, USNM slide 33919, Fraser Mill, British Columbia **86** Oviscape, lateral view (0.5 mm) **87** Genital sclerite, ventral view. (Scale lengths in parentheses).

**Figures 88–96.**
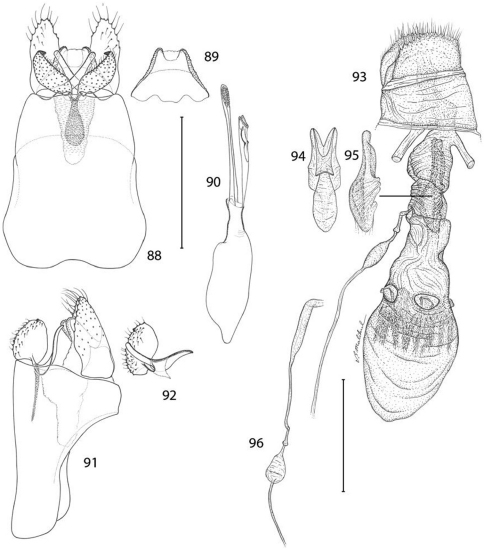
*Epimartyria pardella*, Genitalic morphology **88–92** Male, USNM slide 16613, Arcata, California **88** Genital capsule, ventral view (0.5 mm) **89** Sternum X (gnathos) **90** Aedeagus **91** Genital capsule, lateral view **92** Valva **93–96** Female, DRD slide 4528, Kneeland, California **93** Oviscape, lateral view (0.5 mm) **94** Genital sclerite, ventral view **95** Genital sclerite, lateral view **96** Ductus spermathecae, showing variation of vesicle position. (Scale lengths in parentheses).

## DNA barcoding

A total of 44 specimens yielded barcode sequences, of which 40 were full-length at 658 bp (Appendix 1). Geographic representation of barcoded specimens was primarily dictated by the availability of recently collected material (<20 years), and consequently restricted to a few localities which do not represent the entire range of the species (Fig. 32). Three barcode sequences of *Epimartyria* were available in GenBank, two for *auricrinella* and one for *pardella* (from Lees et al. 2010). The *auricrinella* sequences were identical to haplotype A1 from specimens collected at Gatineau Park, QC, a locality from which a series was examined but not barcoded (see ‘Material examined’ under *auricrinella*). The *pardella* sequence was one base pair different from haplotype P1 from a specimen collected at the same locality although on a different date. These sequences were not included in the analyses because the vouchers could not be examined and their haplotype similarity would not have affected the outcome of the analyses.

Neighbour-joining analysis resulted in three distinct clusters that corresponded to the three species as defined here on the basis of morphology (Fig. 12a). *Epimartyria pardella* was the most distant species with mean distances of 4.52% and 5.09% from *Epimartyria bimaculella* and *Epimartyria auricrinella*, respectively. Morphologically it is the most distinct of the three species in genitalia. *Epimartyria auricrinella* and *Epimartyria bimaculella* seemed to be genetically closer to each other, with a mean distance of 2.57%. Morphologically, their genitalia are also more similar. Intraspecific sequence variation was small in *Epimartyria bimaculella* at 0.2% ± 0.1 and a minimum of three haplotypes could be distinguished. Two specimens with either short sequences or ambiguous sites were not assigned as haplotypes. In contrast, *Epimartyria auricrinella* showed a high amount of sequence divergence resulting in 7 subclusters representing different haplotypes. Pairwise divergence ranged from 0.16–2.69% (1.63% ± 0.832), with 9 out of 21 comparisons showing over 2% divergence (Table 1).

To assess whether the high amount of intraspecific divergence may be correlated with morphological variation, geography, or both, representatives of each haplotype were further subjected to a parsimony analysis. Maximum-parsimony analysis performed on unique haplotypes (7 for *auricrinella*, 3 for *bimaculella*, 1 for *pardella*) resulted in three most parsimonious trees, of which the strict consensus is illustrated (Fig. 12b). Of the 658 base pairs of the full barcode dataset, 607 were constant and 51 were variable, of which only 25 were parsimony-informative. The MP cladogram was similar to the NJ tree in that the three morphospecies were retained as separate, well-supported clades. Despite high sequence variation within the *auricrinella* clade, support for internal nodes was generally weak.

The haplotype network (Fig. 12c) resulted in a similar topology, with the three species separated from each other. In some cases, several haplotypes were present among specimens from the same locality (Fig. 32). For *bimaculella*, there were three haplotypes with 1–2 base pair differences from the type locality of Belcarra, BC, which were collected microsympatrically, two of which on the same date (Appendix 2). Similarly, for *auricrinella*, four haplotypes were present at Lac Brûlé, QC, of which three were 1–2 base pair apart but one (A5) was more than 10 bp divergent. At that locality haplotypes A1, A2 and A5 were present among specimens collected microsympatrically on the same date on two consecutive years (8 Jul 2002, 29 Jun 2003). Haplotype A4 from Tennessee, was closer to haplotypes A1–A3 from Lac Brûlé than to A5 from the same locality. The two localities are over 1300 km apart. Haplotypes A6 and A7 from Michigan were the most divergent, despite being geographically closer to Lac Brûlé than to Tennessee. The majority of barcode sequences came from a single locality for each of *auricrinella* and *bimaculella*. Thus it appears that higher haplotype diversity is associated with denser barcode sampling at single localities.

Genitalia were examined in several specimens of *auricrinella* representing the different haplotypes (Appendix 2). This showed slight variation in the shape of the male valva, in which the inner margin varied from evenly rounded to medially angulate (Fig. 78b–d). Several specimens showed various intermediate states of this condition from having a barely suggested median angle to a sharp one. The angulation differed slightly between the two valvae on some specimens. Likewise slight variation was observed in the depth of the apical notch of the uncus, which was a little deeper or a little more sharply V-shaped in some whereas it was proportionally shallower and more obtusely V-shaped in others. The lateral dentation and recurved distal lobes of the gnathos also displayed minor variations. The variation observed in male genitalia was present across haplotypes from the same locality and seemed unrelated to geography. Males were predominant in all series examined, thus fewer females were compared. No detectable variation was observed in the latter.

Thus we consider the variation in both haplotypes and morphology to be intraspecific. Although a 2% minimal divergence threshold is commonly observed to separate species, and in particular Lepidoptera (Hebert et al. 2009), this rule of thumb is not universal and instances of high intraspecific divergence (Schmidt and Sperling 2008) or shared haplotypes among closely related species (Lumley and Sperling 2010) are known. It has been hypothesized that geographical isolation is likely a major factor in the speciation and diversification of Micropterigidae due to their low vagility, narrow habitat requirement, and frequent allopatry (Imada et al. 2011). Further study involving much broader haplotype sampling of mtDNA and possibly nuclear genes will be required to elucidate the genetic structure of *Epimartyria* populations and whether cryptic species may be present.

**Table 1.** Percent mitochondrial cytochrome c oxidase I (COI) sequence divergence among 11 unique haplotypes of three *Epimartyria* species. Cells below diagonal = distances in %; cells above diagonal = standard error estimates.

	**A1**	**A2**	**A3**	**A4**	**A5**	**A6**	**A7**	**B1**	**B2**	**B3**	**P1**
*Epimartyria auricrinella* A1		0.15	0.26	0.36	0.49	0.57	0.52	0.62	0.60	0.60	0.78
*Epimartyria auricrinella* A2	0.16		0.21	0.33	0.51	0.59	0.54	0.60	0.58	0.58	0.78
*Epimartyria auricrinella* A3	0.47	0.31		0.40	0.57	0.64	0.59	0.65	0.64	0.63	0.82
*Epimartyria auricrinella* A4	0.94	0.78	1.10		0.57	0.59	0.59	0.63	0.62	0.62	0.81
*Epimartyria auricrinella* A5	1.73	1.89	2.21	2.37		0.49	0.50	0.59	0.57	0.59	0.84
*Epimartyria auricrinella* A6	2.20	2.37	2.69	2.53	1.73		0.21	0.69	0.67	0.67	0.89
*Epimartyria auricrinella* A7	1.88	2.04	2.37	2.53	1.73	0.31		0.65	0.63	0.63	0.87
*Epimartyria bimaculella* B1	2.53	2.37	2.69	2.69	2.37	3.18	2.85		0.14	0.21	0.79
*Epimartyria bimaculella* B2	2.37	2.20	2.53	2.53	2.21	3.01	2.69	0.16		0.15	0.78
*Epimartyria bimaculella* B3	2.36	2.20	2.52	2.52	2.37	3.01	2.69	0.31	0.15		0.78
*Epimartyria pardella* P1	4.80	4.80	5.14	5.14	5.14	5.47	5.13	4.63	4.47	4.47	

## Supplementary Material

XML Treatment for Epimartyria

XML Treatment for Epimartyria auricrinella

XML Treatment for Epimartyria bimaculella

XML Treatment for Epimartyria pardella

## Appendix 1

### Appendix 1

**Table:** Sample information for the *Epimartyria* specimens included in the DNA barcode analysis. Sample IDs are specimen identifiers; Barcode IDs (or Process IDs in BOLD) are sequence identifiers. Details of collecting data, images, sequences, and trace files for the 44 specimens listed are available in the Barcode of Life Database (BOLD) (www.barcodinglife.org) in the project codes indicated.

Species of *Epimartyria*	Locality	Sample ID	Barcode ID	GenBank Accession	Sequence Length	BOLD Project Code
*Epimartyria auricrinella*	Canada: QC: lac Brûlé	CNCLEP00002816	MNAC969-11	JN306512	658[0n]	ZEUNA
*Epimartyria auricrinella*	Canada: QC: lac Brûlé	CNCLEP00002817	MNAC970-11	JN306513	658[0n]	ZEUNA
*Epimartyria auricrinella*	Canada: QC: lac Brûlé	CNCLEP00002818	MNAC971-11	JN306514	658[0n]	ZEUNA
*Epimartyria auricrinella*	Canada: QC: lac Brûlé	CNCLEP00002819	MNAC972-11	JN306515	658[0n]	ZEUNA
*Epimartyria auricrinella*	Canada: QC: lac Brûlé	CNCLEP00007712	MNAC974-11	JN306516	658[0n]	ZEUNA
*Epimartyria auricrinella*	Canada: QC: lac Brûlé	CNCLEP00007713	MNAC975-11	JN306517	658[0n]	ZEUNA
*Epimartyria auricrinella*	Canada: QC: lac Brûlé	CNCLEP00007714	MNAC976-11	JN306518	658[0n]	ZEUNA
*Epimartyria auricrinella*	Canada: QC: lac Brûlé	CNCLEP00007715	MNAC977-11	JN306519	658[0n]	ZEUNA
*Epimartyria auricrinella*	Canada: QC: lac Brûlé	CNCLEP00007716	MNAC978-11	JN306520	658[0n]	ZEUNA
*Epimartyria auricrinella*	Canada: QC: lac Brûlé	CNCLEP00007717	MNAC979-11	JN306521	658[0n]	ZEUNA
*Epimartyria auricrinella*	Canada: QC: lac Brûlé	CNCLEP00007718	MNAC980-11	JN306522	658[0n]	ZEUNA
*Epimartyria auricrinella*	Canada: QC: lac Brûlé	CNCLEP00007719	MNAC981-11	JN306523	648[0n]	ZEUNA
*Epimartyria auricrinella*	Canada: QC: lac Brûlé	CNCLEP00007720	MNAC982-11	JN306524	658[0n]	ZEUNA
*Epimartyria auricrinella*	USA: MI: Wilderness SP	CNCLEP00068782	MNAC984-11	JN306525	585[0n]	ZEUNA
*Epimartyria auricrinella*	USA: MI: Wilderness SP	CNCLEP00068783	MNAC985-11	JN306526	658[0n]	ZEUNA
*Epimartyria auricrinella*	USA: MI: Wilderness SP	CNCLEP00068785	MNAC987-11	JN306527	658[0n]	ZEUNA
*Epimartyria auricrinella*	Canada: QC: lac Brûlé	CNCLEP00068787	MNAC989-11	JN306528	658[0n]	ZEUNA
*Epimartyria auricrinella*	Canada: QC: lac Brûlé	CNCLEP00068788	MNAC990-11	JN306529	658[0n]	ZEUNA
*Epimartyria auricrinella*	USA: TN: UoTennessee Field Stn	DNA-ATBI-3323	LGSMD549-05	GU088371	658[0n]	LGSMD
*Epimartyria auricrinella*	Canada: QC: lac Brûlé	jflandry0031	MEC031-04	GU095823	628[1n]	MEC
*Epimartyria auricrinella*	Canada: QC: lac Brûlé	jflandry0725	MEC725-04	GU095821	658[0n]	MEC
*Epimartyria auricrinella*	Canada: QC: lac Brûlé	jflandry0726	MEC726-04	GU095822	658[0n]	MEC
*Epimartyria bimaculella*	Canada: BC: Glacier NP	10BBCLP-2914	BBLPD916-10	JN801469	658[0n]	ZEUNA
*Epimartyria bimaculella*	Canada: BC: Belcarra Pk	CNCLEP00067716	MNAJ565-09	GU693563	658[0n]	ZEUNA
*Epimartyria bimaculella*	Canada: BC: Belcarra Pk	CNCLEP00076632	MNAC991-11	JN306530	658[0n]	ZEUNA
*Epimartyria bimaculella*	Canada: BC: Belcarra Pk	CNCLEP00076633	MNAC992-11	JN306531	658[0n]	ZEUNA
*Epimartyria bimaculella*	Canada: BC: Belcarra Pk	CNCLEP00076634	MNAC993-11	JN306532	658[0n]	ZEUNA
*Epimartyria bimaculella*	Canada: BC: Belcarra Pk	CNCLEP00076635	MNAC994-11	JN306533	658[0n]	ZEUNA
*Epimartyria bimaculella*	Canada: BC: Belcarra Pk	CNCLEP00076636	MNAC995-11	JN306534	658[0n]	ZEUNA
*Epimartyria bimaculella*	Canada: BC: Belcarra Pk	CNCLEP00076637	MNAC996-11	JN306535	658[0n]	ZEUNA
*Epimartyria bimaculella*	Canada: BC: Belcarra Pk	CNCLEP00076638	MNAC997-11	JN306536	658[0n]	ZEUNA
*Epimartyria bimaculella*	Canada: BC: Belcarra Pk	CNCLEP00076639	MNAC998-11	JN306537	658[0n]	ZEUNA
*Epimartyria bimaculella*	Canada: BC: Belcarra Pk	CNCLEP00076640	MNAC999-11	JN306538	658[0n]	ZEUNA
*Epimartyria bimaculella*	Canada: BC: Belcarra Pk	CNCLEP00076641	MNAC1000-11	JN306509	658[0n]	ZEUNA
*Epimartyria bimaculella*	Canada: BC: Belcarra Pk	CNCLEP00077846	MNAL648-10	HQ965294	658[1n]	ZEUNA
*Epimartyria bimaculella*	Canada: BC: Belcarra Pk	CNCLEP00077847	MNAL649-10	HQ965295	658[0n]	ZEUNA
*Epimartyria bimaculella*	Canada: BC: Belcarra Pk	CNCLEP00077848	MNAL650-10	HQ965296	658[0n]	ZEUNA
*Epimartyria bimaculella*	Canada: BC: Belcarra Pk	CNCLEP00077849	MNAL651-10	JN801470	315[0n]	ZEUNA
*Epimartyria bimaculella*	Canada: BC: Belcarra Pk	CNCLEP00077850	MNAL652-10	HQ965297	658[0n]	ZEUNA
*Epimartyria bimaculella*	Canada: BC: Belcarra Pk	CNCLEP00077851	MNAL653-10	JN801471	658[12n]	ZEUNA
*Epimartyria bimaculella*	Canada: BC: Belcarra Pk	CNCLEP00077852	MNAL654-10	HQ965298	658[0n]	ZEUNA
*Epimartyria bimaculella*	Canada: BC: Belcarra Pk	CNCLEP00077853	MNAL655-10	HQ965299	658[0n]	ZEUNA
*Epimartyria bimaculella*	USA: WA: 14.5 km E Skykomish	CNCLEP00082605	MNAD991-11	JN801472	658[0n]	ZEUNA
*Epimartyria pardella*	USA: CA: Kneeland	DRD-08-4201	NAMUM303-08	JN801473	658[0n]	ZEUNA

### Appendix 2

**Table:** Specimen data for haplotype analysis of *Epimartyria*. Haplotypes marked with an asterisk were selected for the parsimony analysis.

Species	Hap	SampleID	Length	Dissection	Sex	Locality	Collecting Date
*Epimartyria auricrinella*	A1	CNCLEP00002817	658		M	QC: lac Brûlé	29-Jun-2003
*Epimartyria auricrinella*	A1	CNCLEP00002818	658		M	QC: lac Brûlé	29-Jun-2003
*Epimartyria auricrinella*	A1	CNCLEP00002819	658		M	QC: lac Brûlé	29-Jun-2003
*Epimartyria auricrinella*	A1	CNCLEP00007713	658	MIC 5758	F	QC: lac Brûlé	08-Jul-2002
*Epimartyria auricrinella*	A1*	CNCLEP00007714	658	MIC 5762	M	QC: lac Brûlé	08-Jul-2002
*Epimartyria auricrinella*	A1	CNCLEP00007715	658		M	QC: lac Brûlé	08-Jul-2002
*Epimartyria auricrinella*	A1	CNCLEP00007716	658		F	QC: lac Brûlé	08-Jul-2002
*Epimartyria auricrinella*	A1	CNCLEP00007717	658	MIC 5763	F	QC: lac Brûlé	08-Jul-2002
*Epimartyria auricrinella*	A1	CNCLEP00007720	658	MIC 5757	M	QC: lac Brûlé	08-Jul-2002
*Epimartyria auricrinella*	A1	CNCLEP00068787	658		F	QC: lac Brûlé	02-Jul-2000
*Epimartyria auricrinella*	A1	jflandry0726	658	USNM 34322	M	QC: lac Brûlé	04-Jul-2004
*Epimartyria auricrinella*	A2*	CNCLEP00007712	658	MIC 5755	M	QC: lac Brûlé	08-Jul-2002
*Epimartyria auricrinella*	A3*	CNCLEP00068788	658	MIC 5756	M	QC: lac Brûlé	02-Jul-2000
*Epimartyria auricrinella*	A4*	DNA-ATBI-3323	658	MIC 5759	F	TN: UoTN stn	22-May-2005
*Epimartyria auricrinella*	A5	CNCLEP00002816	658		M	QC: lac Brûlé	29-Jun-2003
*Epimartyria auricrinella*	A5*	CNCLEP00007718	658	MIC 5753	M	QC: lac Brûlé	08-Jul-2002
*Epimartyria auricrinella*	A5	jflandry0725	658	MIC 5754	F	QC: lac Brûlé	04-Jul-2004
*Epimartyria auricrinella*	A5?	CNCLEP00007719	648	MIC 5760	M	QC: lac Brûlé	08-Jul-2002
*Epimartyria auricrinella*	A5?	jflandry0031	628 [1n]	MIC 5761	M	QC: lac Brûlé	29-Jun-2003
*Epimartyria auricrinella*	A6*	CNCLEP00068783	658	MIC 5752	M	MI: Wilderness SP	30-Jun-1992
*Epimartyria auricrinella*	A6?	CNCLEP00068782	585		M	MI: Wilderness SP	30-Jun-1992
*Epimartyria auricrinella*	A7*	CNCLEP00068785	658	MIC 5764	M	MI: Wilderness SP	30-Jun-1992
*Epimartyria bimaculella*	B1	CNCLEP00076633	658		M	BC: Belcarra	24-May-2009
*Epimartyria bimaculella*	B1	CNCLEP00076635	658	MIC 5771	F	BC: Belcarra	01-Jun-2009
*Epimartyria bimaculella*	B1	CNCLEP00076638	658		M	BC: Belcarra	01-Jun-2009
*Epimartyria bimaculella*	B1	CNCLEP00076639	658	MIC 5767	M	BC: Belcarra	01-Jun-2009
*Epimartyria bimaculella*	B1	CNCLEP00076640	658		M	BC: Belcarra	02-Jun-2009
*Epimartyria bimaculella*	B1	CNCLEP00077846	658		M	BC: Belcarra	01-Jun-2009
*Epimartyria bimaculella*	B1*	CNCLEP00077847	658	MIC 5765	M	BC: Belcarra	01-Jun-2009
*Epimartyria bimaculella*	B1	CNCLEP00077848	658		M	BC: Belcarra	01-Jun-2009
*Epimartyria bimaculella*	B1	CNCLEP00077852	658		M	BC: Belcarra	01-Jun-2009
*Epimartyria bimaculella*	B2	10BBCLP-2914	658	MIC 5769	F	BC: Glacier NP	16-Jul-2010
*Epimartyria bimaculella*	B2	CNCLEP00076632	658		M	BC: Belcarra	24-May-2009
*Epimartyria bimaculella*	B2	CNCLEP00076634	658	MIC 5770	M	BC: Belcarra	01-Jun-2009
*Epimartyria bimaculella*	B2	CNCLEP00076636	658		M	BC: Belcarra	01-Jun-2009
*Epimartyria bimaculella*	B2	CNCLEP00076637	658		M	BC: Belcarra	01-Jun-2009
*Epimartyria bimaculella*	B2	CNCLEP00076641	658		M	BC: Belcarra	02-Jun-2009
*Epimartyria bimaculella*	B2	CNCLEP00077850	658		M	BC: Belcarra	01-Jun-2009
*Epimartyria bimaculella*	B2	CNCLEP00077853	658		M	BC: Belcarra	01-Jun-2009
*Epimartyria bimaculella*	B2*	CNCLEP00082605	658	MIC 5739	M	WA: Skykomish	08-Jul-2010
*Epimartyria bimaculella*	B3*	CNCLEP00067716	658	MIC 5768	M	BC: Belcarra	08-Jun-2008
*Epimartyria bimaculella*	B?	CNCLEP00077849	315		M	BC: Belcarra	01-Jun-2009
*Epimartyria bimaculella*	B?	CNCLEP00077851	658[12n]	MIC 5766	M	BC: Belcarra	01-Jun-2009
*Epimartyria pardella*	P1*	DRD-08-4201	658		?	CA: Kneeland	21-Jun-2008
